# Synthesis and characterization of pH responsive D-glucosamine based molecular gelators

**DOI:** 10.3762/bjoc.10.328

**Published:** 2014-12-23

**Authors:** Navneet Goyal, Hari P R Mangunuru, Bargav Parikh, Sonu Shrestha, Guijun Wang

**Affiliations:** 1 Department of Chemistry and Biochemistry, Old Dominion University, 4541 Hampton Boulevard, Norfolk, VA 23529, USA

**Keywords:** glucosamine, hydrogelators, naproxen, organogelator, pH responsive, self-assembly

## Abstract

Small molecular gelators are a class of compounds with potential applications for soft biomaterials. Low molecular weight hydrogelators are especially useful for exploring biomedical applications. Previously, we found that 4,6-*O*-benzylidene acetal protected D-glucose and D-glucosamine are well-suited as building blocks for the construction of low molecular weight gelators. To better understand the scope of D-glucosamine derivatives as gelators, we synthesized and screened a novel class of *N*-acetylglucosamine derivatives with a *p*-methoxybenzylidene acetal protective group. This modification did not exert a negative influence on the gelation. On the contrary, it actually enhanced the gelation tendency for many derivatives. The introduction of the additional methoxy group on the phenyl ring led to low molecular weight gelators with a higher pH responsiveness. The resulting gels were stable at neutral pH values but degraded in an acidic environment. The release profiles of naproxen from the pH responsive gels were also analyzed under acidic and neutral conditions. Our findings are useful for the design of novel triggered release self-assembling systems and can provide an insight into the influence of the the structure on gelation.

## Introduction

Low molecular weight gelators (LMWGs) have drawn great attention over the past few decades due to the formation of supramolecular structures and their potential applications as advanced materials [1–7]. LMWGs are also referred to as supramolecular gelators or molecular gelators. They can form reversible gels in organic solvents, aqueous mixtures, and water. The gelation process is completely driven by weak intermolecular forces such as hydrogen bonding, π–π stacking, hydrophobic forces, and van der Waals forces. The collective weak interactions of gelators result in self-assembled supramolecular networks and lead to the formation of stable reversible gels. A majority of small molecular gelators were discovered by chance and their structural requirements are ambiguous. Many different structural classes have been found to be effective LMWGs, including derivatives from amino acids, carbohydrates, and cholesterols derivatives [8–16]. The reversible organogels and hydrogels have been explored for many applications including drug delivery, protein binding and separation, tissue engineering, and the controlled release of certain biological agents [17–27].

**Figure 1:**
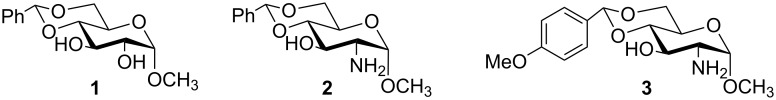
General structures of several D-glucose and D-glucosamine derivatives.

Molecular gelators containing photo or pH responsive functional groups are able to form multi-stimuli responsive gels. These resulting gels may find applications as advanced soft functional materials [28]. Several pH responsive small molecular gelators have been designed and synthesized and have shown a variety of potential applications [29–34]. Among the different classes of LMWGs, carbohydrate-based systems are especially interesting due to their potential applications in biomedical research. Furthermore, they may be derived from abundant renewable resources. We have studied the selective functionalization of monosaccharides, such as glucose and glucosamine, and obtained effective low molecular weight gelators for both organic solvents and aqueous mixtures [35–40]. Previously we mostly focused on the modification of the C-2 position of the benzylidene acetal protected headgroups **1** and **2** (Figure 1). We obtained the general structural requirements for acyl derivatives at the 2-position. In this study, we explore the substituent effect at the benzylidene acetal protective group by introducing an electron donating *p*-methoxy functional group and form the headgroup **3**. By synthesizing similar acyl derivatives used for the functionalization of compound **2**, we can analyze whether the introduction of the *p*-methoxy group affects the gelation. The probing of the structural scope of the headgroup of this class of LMWGs lays the basis for the design of organo/hydrogelators with desired functionalities. The *p*-methoxybenzylidene acetal is more pH responsive in comparison to the benzylidene acetal protective group. The *p*-methoxybenzylidene acetal can be cleaved in the presence of acids much more readily which results in pH responsive triggered release organogel or hydrogels, provided that the derivatives of compound **3** are effective gelators.

## Results and Discussion

**Scheme 1:**
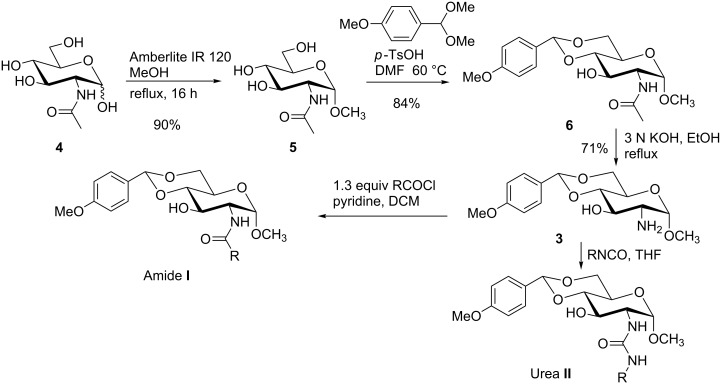
Synthesis of amide and urea derivatives of the headgroup **3**.

In order to understand the scope of functionalization on monosaccharides and the structure influence of the benzylidene acetal group, we synthesized a small library of amide and urea derivatives of head group **3** (Scheme 1) and screened their gelation properties. Similar solvent systems used for our previous small molecular gelators were used, and selected representative hydrophobic functional groups were studied. We expect that other C-2 analogs with similar polarities exhibit similar self-assembling properties. Scheme 1 shows the preparation of the headgroup **3**, the amide **I** and urea **II** analogs by a similar method as reported previously [36].

**Figure 2:**
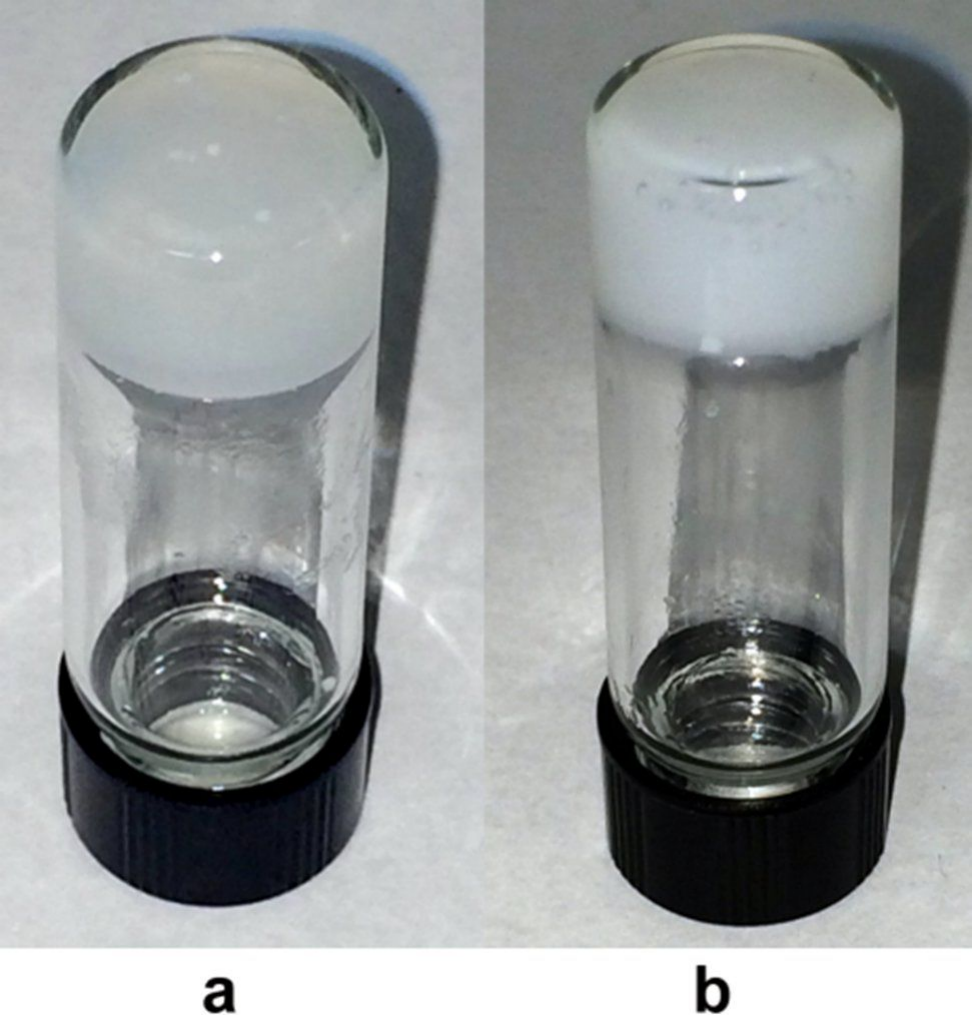
a) A translucent/opaque gel formed by compound **19** in EtOH/H_2_O (1:2) at 1.3 mg/mL; b) a gel formed by compound **16** in EtOH/H_2_O (1:2) at 2.0 mg/mL.

Several alkyl and aryl derivatives are synthesized for the amide and urea substituted **3**. These include the short chain alkyl, phenyl, and naphthyl groups. The gelation properties of the amide and urea derivatives are shown in Table 1. We found that nearly all analogs synthesized and screened are effective gelators for ethanol, ethanol/water (1:2 by volume), and DMSO/water (1:2 by volume) mixtures. Several amides are also effective gelators for water. For this series of compounds, the amides seem to be more effective for water, whereas the ureas are more effective for ethanol and aqueous mixtures. These results indicate that the additional *p*-methoxy group did not affect gelation negatively. Instead, it may actually enhance gelation tendencies. The gels are typically translucent to opaque. Two photographs of typical gels are shown in Figure 2.

**Table 1:** Gelation test results for amide derivatives **I** (**6–14**) and urea derivatives **II** (**15–22**).^a^

Compound	Structure	Hexane	Water	EtOH	Water/DMSO (2:1)	Water/EtOH (2:1)

**6**	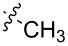	I	G 10.0	S	G 7.4	G 8.0
**7**	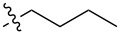	I	I	G 10.0	G 4.0	G 4.0
**8**	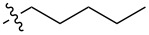	C	C	G 20.0	G 5.4	G 6.0
**9**	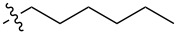	I	I	G 20.0	G 5.7	G 5.7
**10**	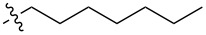	I	I	G 10.0	G 4.6	G 8.0
**11**	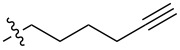	C	C	G 20.0	G 5.0	G 8.8
**12**	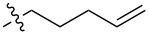	C	I	S	G 6.0	G 7.4
**13**	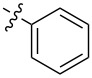	I	G 2.0	G 8.0	G 5.7	G 10.0
**14**	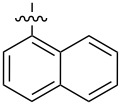	C	I	G 10.0	G 20.0	G 10.0
**15**	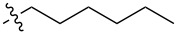	I	I	G 10.0	G 4.0	G 2.0
**16**	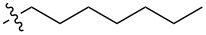	I	P	G 5.0	G 2.0	G 2.0
**17**	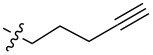	P	I	G 5.0	G 4.0	G 3.3
**18**	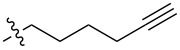	C	I	G 10.0	G 5.0	G 5.0
**19**	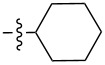	I	I	G 2.5	G 2.0	G 1.3
**20**	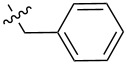	P	I	G 3.3	G 4.0	G 4.0
**21**	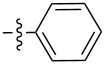	I	I	G 6.6	G 2.8	G 5.0
**22**	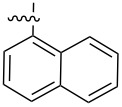	I	I	G 20.0	G 4.0	G 5.0

^a^G, stable gel at room temperature, the number after G is the minimum gelation concentration (MGC) in mg/mL; I, insoluble; C, crystallization; P, precipitate; S, soluble at 20 mg/mL.

**Figure 3:**
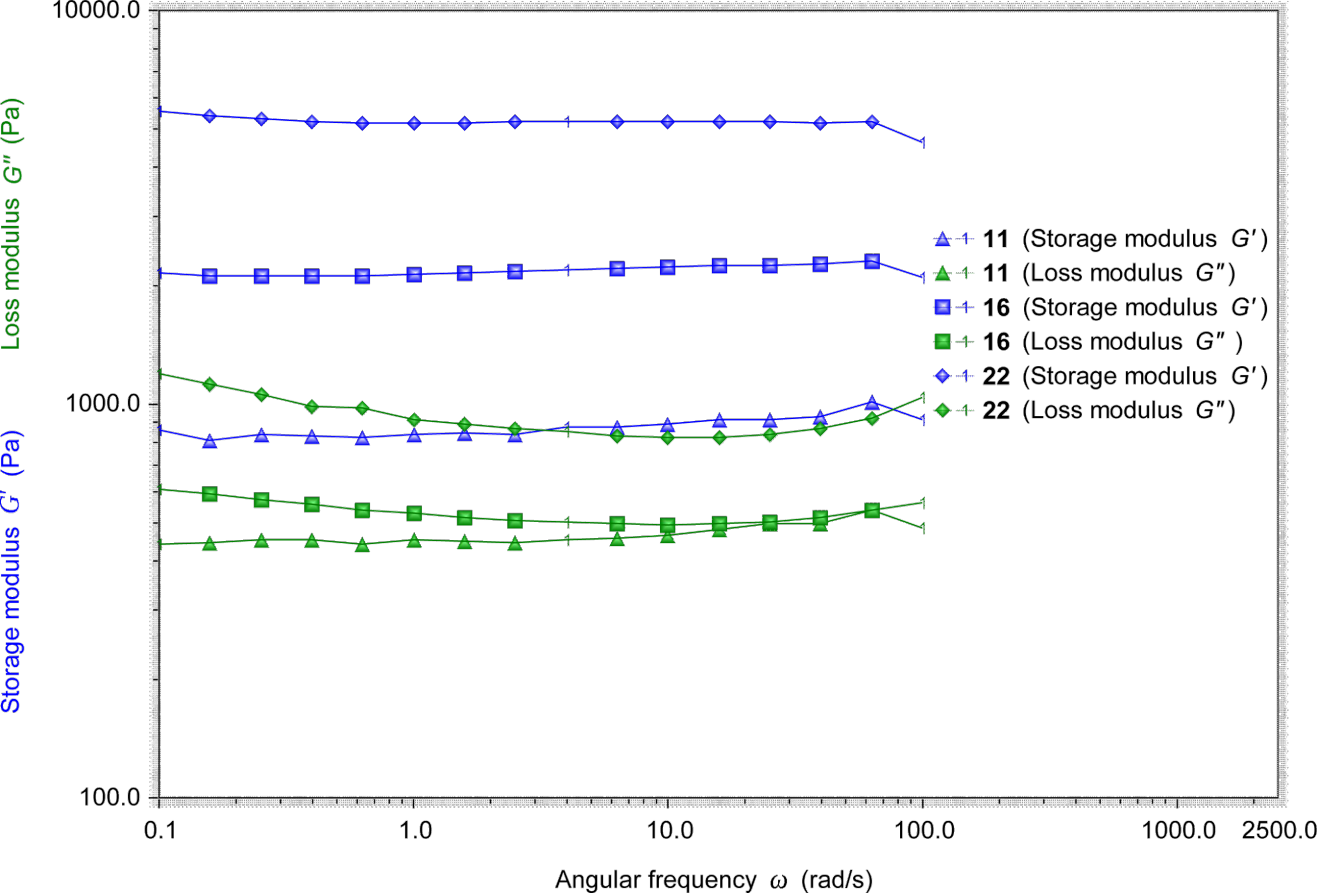
The rheological measurements of the gels formed by amide **11** (EtOH/H_2_O, 1:2) at 5.4 mg/mL, urea **16** (DMSO/H_2_O, 1:2) at 2.0 mg/mL, and urea **22** (DMSO/H_2_O, 1:2) at 4.0 mg/mL.

We then studied the stability and elastic properties of several gels by using a rheometer. Selected results are shown in Figure 3. For all the tested gels at the frequency sweep, the storage modulus *G'* is always greater than the loss modulus *G''*. They are mostly independent of the dynamic frequencies, which is an indication of the gel’s stability and elasticity. Among the few gels tested, the storage modulus *G'* of the gel formed by urea **17** (in DMSO/H_2_O, 1:2, 4.0 mg/mL) is about 14000 Pa, and the loss modulus *G''* is about 3500 Pa (Figure S1 in Supporting Information File 1). For the gel formed by urea **22** in DMSO/H_2_O, its *G'* value is about 5000 Pa and *G''* is about 1000 Pa. The gel formed by urea **16** showed a *G'* value of about 2000 Pa and *G''* of about 500 Pa. The gel formed by amide **11** had smaller *G'* and *G''* values. These results indicate that all the gels are stable and have elastic properties. The gels formed by urea derivatives are more stable than the amide gels.

**Figure 4:**
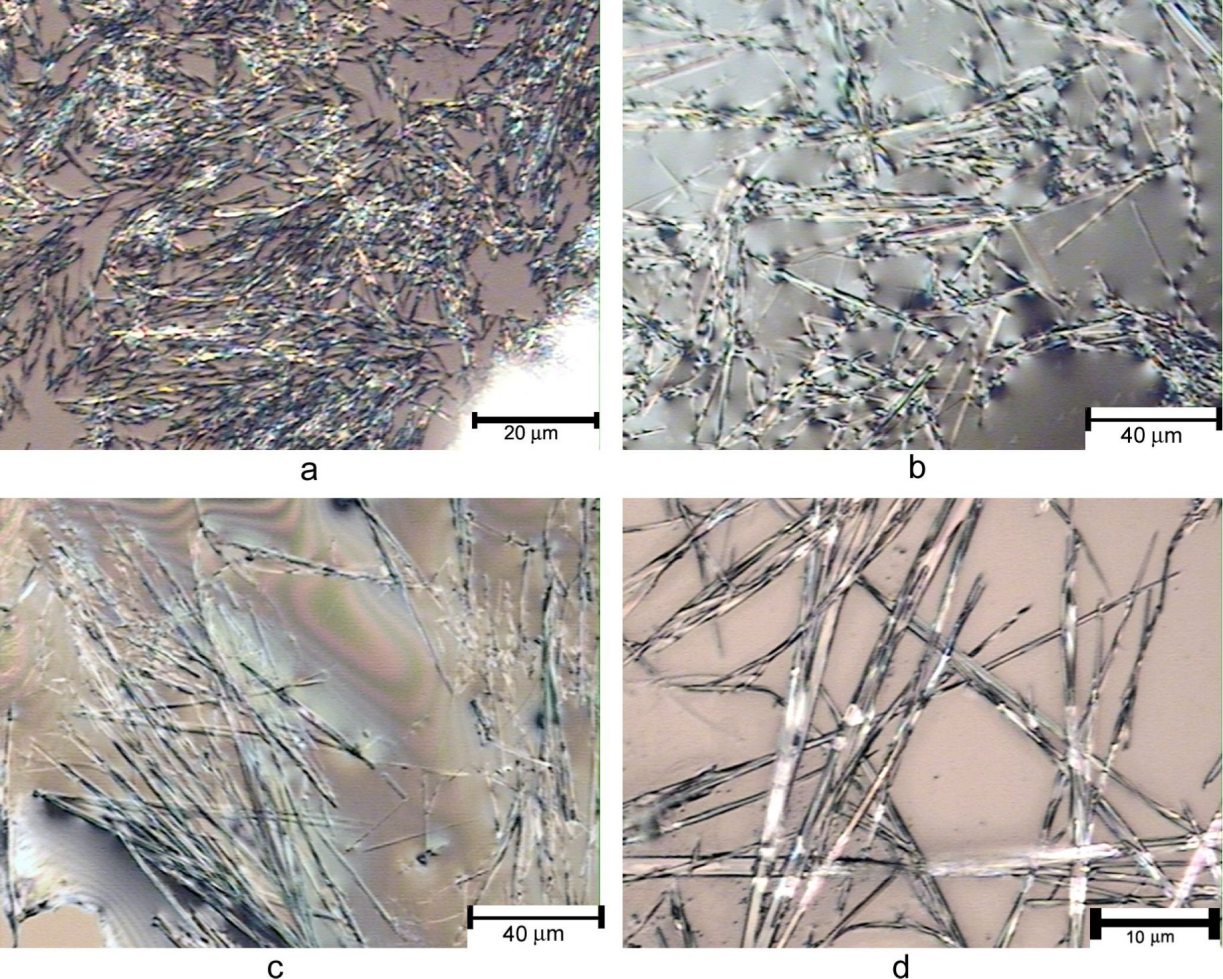
The optical micrographs of the wet gels formed by several amides. a) A gel formed by compound **8** in EtOH/H_2_O (1:2) at 5.4 mg/mL; b) a gel formed by compound **13** in DMSO/H_2_O (1:2) at 5.7 mg/mL; c) and d) are a gel formed by compound **11** in EtOH/H_2_O (1:2) at 5.4 mg/mL.

The morphology of the amide and urea gels was also studied by optical microscopy. As shown in Figure 4, the gels from the various amide derivatives typically formed a fibrous network, compound **8** formed shorter fibers (~20 μm in lengths), compound **13** formed longer fibrous assemblies (40–100 μm), and compound **11** formed long and uniform fibers with average lengths over 100 μm.

**Figure 5:**
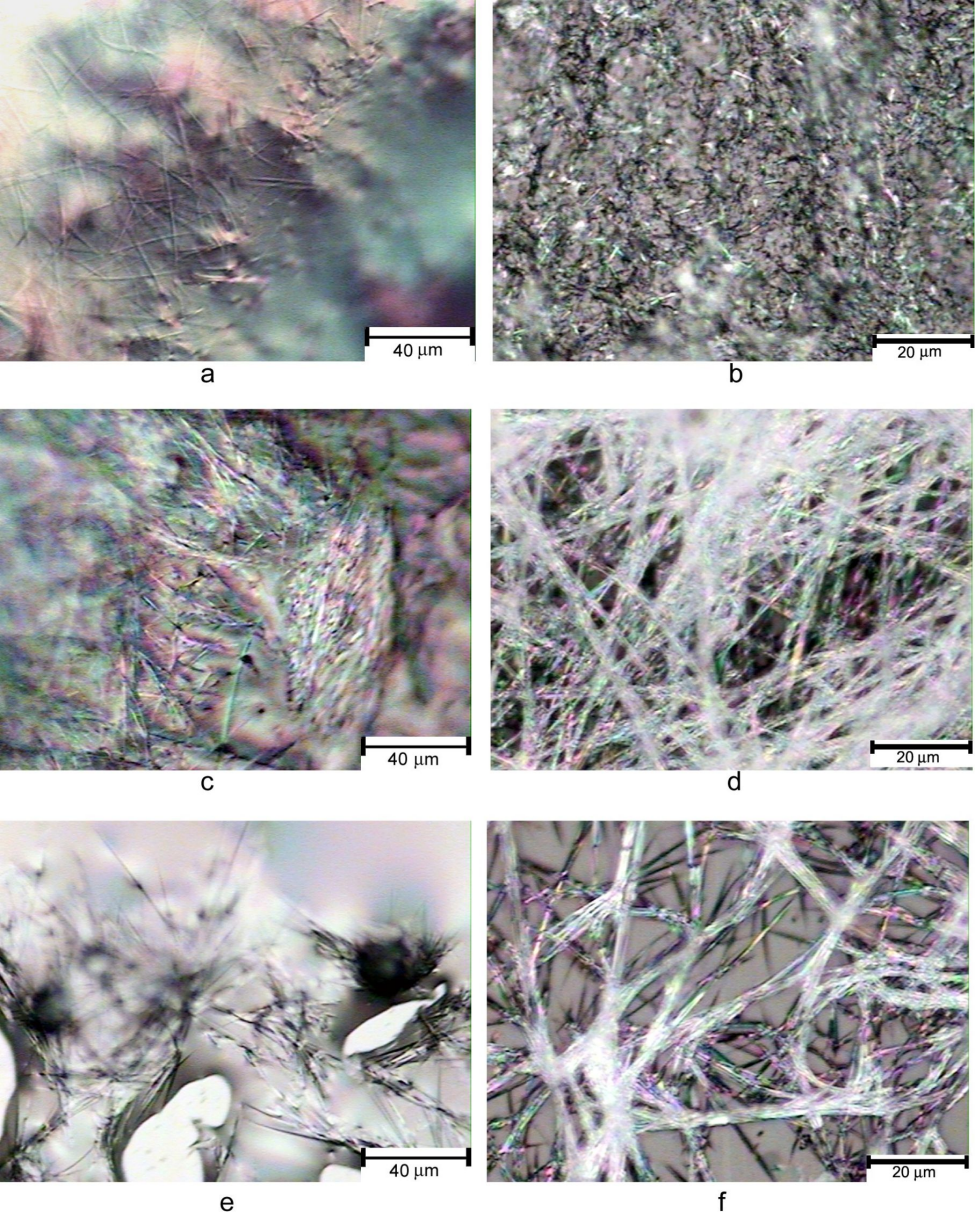
The optical micrographs of the wet gel samples formed by several ureas. a) Compound **19** in DMSO/H_2_O (1:2) at 2.0 mg/mL; b) compound **17** in EtOH/H_2_O (1:2) at 3.3 mg/mL; c) and d) a gel formed by compound **21** in EtOH/H_2_O (1:2) at 5.0 mg/mL; e) and f) are from the gel formed by compound **22** in DMSO/H_2_O (1:2) at 4.0 mg/mL.

The gels formed by the urea derivatives are shown in Figure 5. These samples still contain small amounts of solvent. Compound **19** formed fibrous assemblies in DMSO/H_2_O solvent, **21** formed long intertwined fibrous networks, and the naphthyl urea **22** also formed uniform fibrous assemblies.

**Figure 6:**
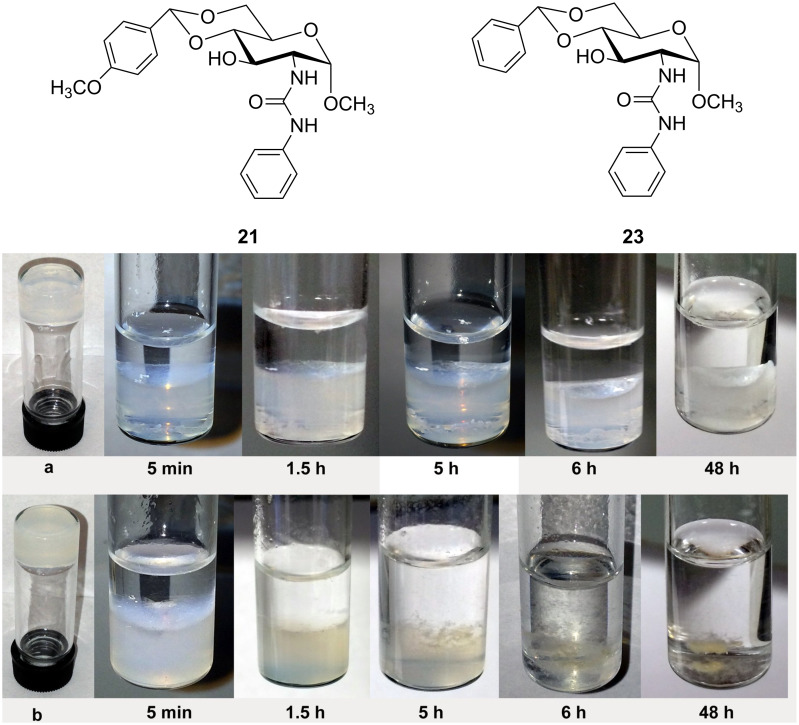
Stability test results under acidic conditions. a) A gel formed by compound **23** in DMSO/H_2_O (1:2) at 5 mg/mL. b) A gel formed by compound **21** in DMSO/H_2_O (1:2) at 5 mg/mL. The photos show the gels treated with 1 mL of sulfuric acid solution (pH 1) at different time periods.

A comparison of the gelation test results in Table 1 and the benzylidene acetal derivatives reported earlier [36] reveals that the introduction of the *p*-methoxy group entails similar or enhanced gelation capabilities (formed gels in lower concentrations). The *p*-OMe derivatives are expected to be more pH responsive compared to the non-substituted series, which may be useful for a triggered release of entrapped agents under an acidic environment. We tested the stability of two aqueous gels formed by compounds **23** [36] and **21** by using diluted sulfuric acid solution (pH 1). As shown in Figure 6, the stability of two gels is analyzed at different time periods in the presence of the acid. The gel formed by the benzylidene acetal **23** was stable after 6 h and no obvious decomposition was observed. After 48 h only a small amount of the gel was dissolved and the gel feature was largely maintained. This indicates that the gel formed by compound **23** is stable under an acidic environment. The gel formed by compound **21** showed a rapid degradation upon treatment with acid. After 5 h exposure to the acid about half of the gel was dissolved and after 6 h the gel was mostly decomposed.

The gel formed by compound **23** was stable for a day or so and still maintained a certain gel integrity after two days, but the gel formed by **21** showed a rapid disappearance of gel features under acid conditions. This difference in response to an acid treatment can be utilized for acid responsive triggered release studies. We then tested the stability of most of the DMSO/water gels toward acid by using diluted hydrochloric acid solution (pH 1). The stability of the gels was monitored every 30 minutes and the time it took for the gels to totally dissolve was recorded, the results of which are shown in Table 2. The stability of the DMSO/H_2_O gels upon the addition of water (pH 7) for compounds **16**, **17**, **20** and **21** was also tested, and the gels were almost the same after 20 h (Figure S2 in Supporting Information File 1). These results indicate that the gels are stable under neutral conditions and can rapidly dissolve in an acidic environment.

**Table 2:** The acid sensitivity of the gels. All gels were tested at their minimum gelation concentrations (MGC).

Compound	Structure	DMSO/H_2_O (1:2) MGC mg/mL	Estimated time for gel dissolution

**8**	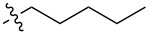	5.4	6 h
**9**	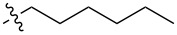	5.7	6 h
**10**	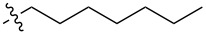	4.6	3.5 h
**13**	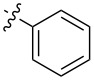	5.7	3.5 h
**15**	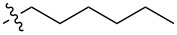	4.0	3 h
**16**	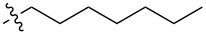	2.0	4 h
**17**	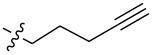	4.0	3.5 h
**18**	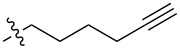	5.0	5 h
**19**	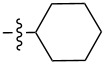	2.0	2 h
**20**	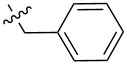	4.0	3 h
**22**	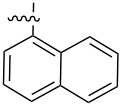	4.0	2.5 h

**Figure 7:**
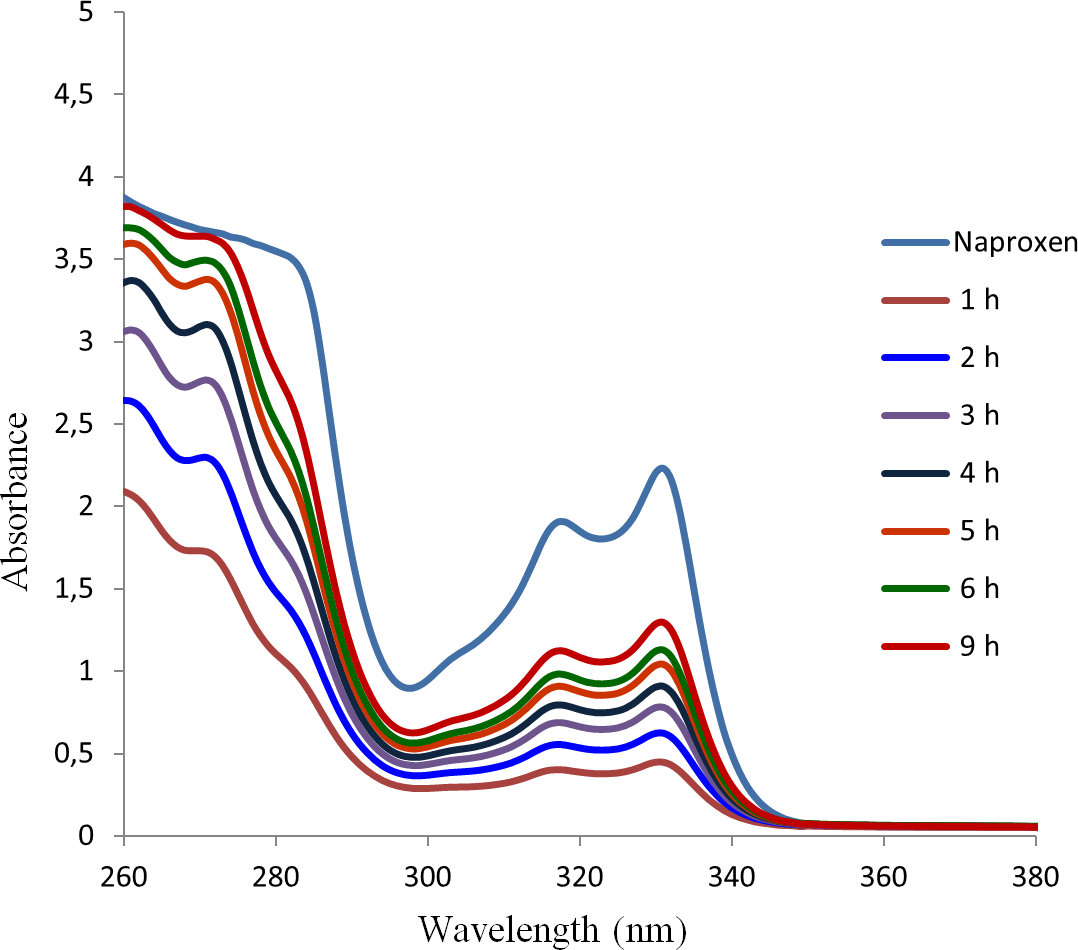
Release of naproxen sodium from the gel formed by compound **16** to the aqueous phase.

**Figure 8:**
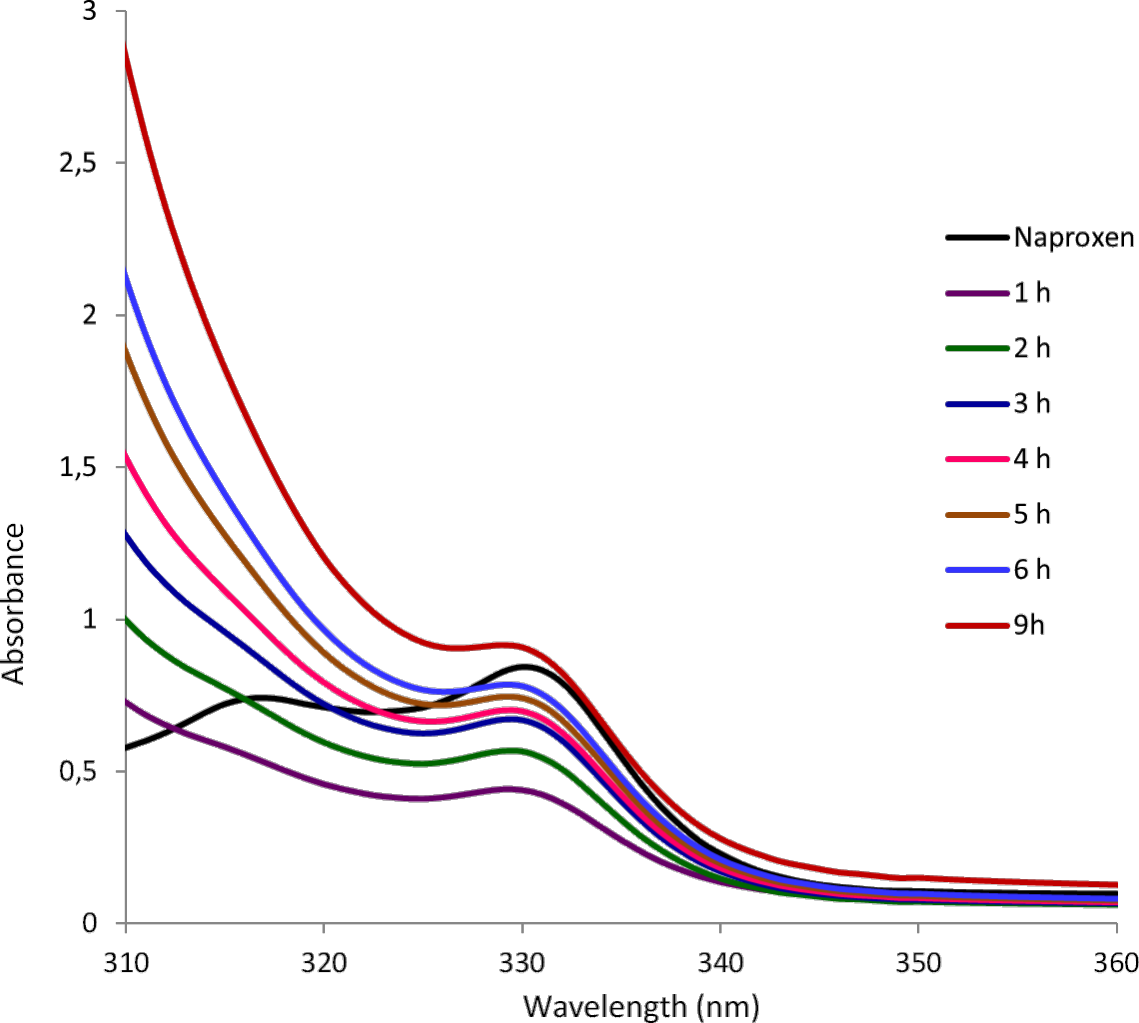
Naproxen release study from the gel formed by compound **16** in the presence of acid.

We anticipated that these types of gels may be useful for the controlled release of drugs or other agents under acidic conditions. We picked the nonsteroidal anti-inflammatory drug (NSAID) naproxen as an example and studied the release profile of the drug trapped in the gel matrix. To test the effectiveness of the trapped drug in the gel matrix, naproxen sodium was incorporated into the gels and the release kinetics of naproxen was monitored with UV spectroscopy. The gel formed by compound **16** in DMSO/water was selected for the study, the release profiles of naproxen from the gel in the presence of water and HCl were monitored at different time intervals. As shown in Figure 7, naproxen was slowly released from the gel phase to the neutral aqueous phase. 1 h after the addition of water, a small amount of naproxen was released from the gel to the aqueous phase, presumably by permeation. The release of naproxen gradually increased with time, after 9 h, about half of the trapped naproxen was released, although the gel mostly remained intact. In contrast, when 0.1 M HCl solution was used instead of water (Figure 8), the naproxen sodium was released much more rapidly. After 9 h naproxen was completely released from the gel. As can be seen from Figure 8, the signals at 330 nm are corresponding to the absorption of naproxen. The gelator molecule **16** reacted rapidly upon addition of the acid, and after 1 h almost half of trapped naproxen was released. After 6 h almost all of the trapped naproxen was released. That the absorption of released naproxen at 9 h is greater than the naproxen control is caused by the *p*-methoxybenzaldehyde generated from the gelator molecule’s decomposition. *p*-Methoxybenzaldehyde exhibits a strong absorption in the wavelength range of 260–320 nm. The UV spectra of the gelator **16** under the same acidic conditions are shown in Figure S3 (Supporting Information File 1), and the full range of UV spectra of the released naproxen are shown in Figure S4, Supporting Information File 1.

## Conclusion

In summary, we have synthesized and studied a new series of D-glucosamine derivatives containing pH responsive functional groups. These amide and urea derivatives are effective low molecular weight gelators. In comparison to the amide gels, the gels formed by the urea derivatives seem to be mechanically stronger and more elastic. The *p*-methoxylbenzylidene acetal protecting group can be cleaved under acidic conditions more easily than the benzylidene acetal group. The pH responsive gelators synthesized are stable under neutral conditions and degrade readily at acidic environment (pH 1–2). Naproxen sodium was entrapped in the gels and the release kinetics were studied. It was released slowly to the aqueous phase under neutral conditions. Under acidic conditions (pH 1.9), on the other hand, naproxen was released rapidly from the gel phase. The compounds reported on herein may be useful for triggered release drug delivery systems under acidic environment.

## Experimental

### Gelation test

In general, about 2 mg of the compounds were transferred to a 1 dram capped vial, to this vial solvents were added in a 0.1 mL increment. The initial concentration was 20 mg/mL (2 mg in 0.1 mL). The mixture was then heated and sonicated to attempt to dissolve the compound. The mixture was then allowed to cool down at room temperature for 15–20 minutes. After this period, if a stable gel was formed serial dilution was performed until the gel became unstable. The gelation concentration prior to the unstable gel was recorded as the Minimum Gelation Concentration (MGC). Otherwise, the observed state of the mixture was recorded.

### Acid stability study

Two gels were prepared in DMSO/H_2_O from compounds **21** and **23** at 5 mg/mL and 1 mL total volume in a small vial. To this gel, 1 mL of sulfuric acid solution (pH 1) was carefully added to the top the gel. This mixture was then monitored at 25 °C to observe the breakage of the gels at different time points. Photos were taken at these time points and recorded. After most of the gels have decomposed the pH of the final solution was 2.3 for both samples.

For the acid sensitivity studies of other compounds, the gels were prepared at their MGCs in DMSO/H_2_O (1:2) for 1 mL in a one dram vial, then 1 mL of HCl solution (pH 1) was carefully added to the top of the gel, the stability was then monitored at 25 °C, and the time for complete gel decomposition was recorded in Table 2.

### Naproxen release study

For naproxen release under neutral conditions (Figure 7), the gel was formed by 6 mg of compound **16** and 1.5 mg of naproxen sodium in 3 mL DMSO/H_2_O (1:2) in a vial. Then 3 mL of water (pH 7.0) was added to the top of the gel, the UV spectrum of the aqueous phase was monitored hourly. The control naproxen graph was obtained using 1.5 mg of naproxen sodium in the same solvent system, 3 mL DMSO/H_2_O (1:2) and 3 mL of water.

For the release of naproxen under acidic conditions (Figure 8), the gel was formed by 6 mg of compound **16** and 1.5 mg of naproxen sodium salt in 3 mL DMSO/H_2_O (1:2). Then 3 mL of 0.1 M HCl (pH 1.9) was carefully added dropwise to the top of the gel. The UV absorbance of the top aqueous phase was monitored at different time intervals, the final pH of the totally dissolved sample was 2.5. The control naproxen graph was obtained using 1.5 mg of naproxen sodium in an identical solvent system, 3 mL DMSO/H_2_O (1:2) and 3 mL of 0.1 M HCl solution, the pH was 2.4 for the control naproxen.

### General procedure for the synthesis of amides

To a 50 mL round bottom flask, 50 mg of head group **3**, dissolved in 2 mL of THF or DCM and 2 equivalents of potassium carbonate or pyridine, were added. The reaction mixture was then cooled to 0 °C and 1 equivalent of the corresponding acid chloride was added dropwise to the solution. The mixture was left stirring for 6–10 h, after which the mixture was concentrated by using a rotary evaporator. The crude residue was purified by flash chromatography by means of a hexane/DCM/MeOH gradient solvent system. The amide derivatives **7–15** were obtained and tested for their gelation properties.

### General procedure for the synthesis of ureas

The urea library was synthesized by mixing compound **3** and the corresponding isocyanate in stoichiometric quantities in anhydrous THF. The solution was stirred at room temperature for 3–5 h. Then the mixture was concentrated by using a rotary evaporator. If needed, the crude products were purified by flash chromatography on silica gel. A DCM/MeOH gradient solvent system was used for the chromatography separation.

### Compound characterization data

**Compound 7:** Pentylamide, isolated as a white solid in 92% yield, mp 200.2–202.0 °C; ^1^H NMR (400 MHz, CDCl_3_) δ 7.44–7.39 (m, 2H), 6.91–6.85 (m, 2H), 5.83 (d, *J* = 8.4 Hz, 1H), 5.52 (s, 1H), 4.71 (d, *J* = 4.0 Hz, 1H), 4.31–4.18 (m, 2H), 3.88 (dt, *J* = 2.6, 9.5 Hz, 1H), 3.80 (s, 3H), 3.79–3.72 (m, 2H), 3.60–3.53 (m, 1H), 3.39 (s, 3H), 3.11 (d, *J* = 2.9 Hz, 1H), 2.25 (t, *J* = 7.3 Hz, 2H), 1.68–1.58 (m, 2H), 1.42–1.30 (m, 2H), 0.92 (t, *J* = 7.3 Hz, 3H); ^13^C NMR (100 MHz, CDCl_3_) δ 174.6, 160.1, 129.6, 127.6, 113.6, 101.7, 98.7, 81.9, 70.6, 68.7, 62.3, 55.2, 54.0, 36.3, 27.6, 22.2, 13.7; HRMS: [M + H]^+^ calcd for C_20_H_29_NO_7_, 396.2021; found, 396.2022.

**Compound 8:** Hexylamide, isolated as a white solid in 87% yield, mp 179.4–180.7 °C; ^1^H NMR (400 MHz, CDCl_3_) δ 7.44–7.39 (m, 2H), 6.91–6.85 (m, 2H), 5.84 (d, *J* = 8.4 Hz, 1H), 5.52 (s, 1H), 4.71 (d, *J* = 4.0 Hz, 1H), 4.29–4.18 (m, 2H), 3.92–3.85 (m, 1H), 3.79 (s, 3H), 3.79–3.71 (m, 2H), 3.61–3.51 (m, 1H), 3.40 (s, 3H), 3.15 (d, *J* = 3.3 Hz, 1H), 2.24 (t, *J* = 7.3 Hz, 2H), 1.69–1.59 (m, 2H), 1.37–1.26 (m, 4H), 0.89 (t, *J* = 7.0 Hz, 3H); ^13^C NMR (100 MHz, CDCl_3_) δ 174.7, 160.2, 129.6, 127.6, 113.6, 101.8, 98.8, 82.0, 70.9, 68.8, 62.3, 55.3, 54.0, 36.6, 31.3, 25.2, 22.3, 13.9; HRMS: [M + H]^+^calcd for C_21_H_32_NO_7_, 410.2177; found, 410.2179.

**Compound 9:** Heptylamide, isolated as a white solid in 88% yield, mp 197.2–198.1 °C; ^1^H NMR (400 MHz, CDCl_3_) δ 7.44–7.39 (m, 2H), 6.91–6.85 (m, 2H), 5.84 (d, *J* = 8.4 Hz, 1H), 5.52 (s, 1H), 4.71 (d, *J* = 3.7 Hz, 1H), 4.30–4.18 (m, 2H), 3.93–3.85 (m, 1H), 3.80 (s, 3H), 3.79–3.73 (m, 2H), 3.61–3.53 (m, 1H), 3.40 (s, 3H), 3.06 (d, *J* = 3.3 Hz, 1H), 2.25 (t, *J* = 7.3 Hz, 2H), 1.71–1.60 (m, 2H), 1.39–1.21 (m, 6H), 0.89 (t, *J* = 7.0 Hz, 2H); ^13^C NMR (100 MHz, CDCl_3_) δ 174.7, 160.2, 129.6, 127.6, 113.6, 101.8, 98.8, 82.0, 70.9, 68.8, 62.3, 55.3, 53.9, 36.5, 31.3, 25.2, 22.3, 13.9; HRMS: [M + H]^+^calcd for C_22_H_34_NO_7_, 424.2331; found, 424.2335.

**Compound 15:** Hexylurea, isolated as a white solid in quantitative yield, mp 206.1–206.9 °C; ^1^H NMR (400 MHz, DMSO) δ 7.40–7.33 (m, 2H), 6.95–6.88 (m, 2H), 6.06 (t, *J* = 5.5 Hz, 1H), 5.77 (d, *J* = 8.4 Hz, 1H), 5.53 (s, 1H), 5.18 (d, *J* = 5.5 Hz, 1H), 4.61 (d, *J* = 3.7 Hz, 1H), 4.14 (dd, *J* = 4.8, 9.9 Hz, 1H), 3.75 (s, 3H), 3.73–3.64 (m, 2 H), 3.61–3.52 (m, 1H), 3.52–3.40 (m, 2H), 3.30 (s, 3H), 3.04–2.90 (m, 2H), 1.40–1.31 (m, 2H), 1.30–1.15 (m, 6H), 0.86 (t, *J* = 6.6 Hz, 3H); ^13^C NMR (100 MHz, DMSO) δ 159.5, 157.9, 130.1, 127.7, 113.2, 100.8, 99.5, 81.9, 68.5, 68.0, 62.5, 55.0, 54.7, 54.6, 39.2, 31.0, 29.9, 26.0, 22.0, 13.8; HRMS: [M + H]^+^calcd for C_22_H_35_N_2_O_7_; 439.2444; found, 459.2445.

**Compound 16:** Heptylurea, isolated as a white solid in quantitative yield, mp 204.2–205.0 °C; ^1^H NMR (400 MHz, DMSO) δ 7.39–7.34 (m, 2H), 6.94–6.89 (m, 2H), 6.06 (t, *J* = 5.5 Hz, 1H), 5.76 (d, *J* = 8.4 Hz, 1H), 5.53 (s, 1H), 5.18 (d, *J* = 5.5 Hz, 1H), 4.61 (d, *J* = 3.7 Hz, 1H), 4.14 (dd, *J* = 4.8, 9.8 Hz, 1H), 3.74 (s, 3H), 3.72–3.64 (m, 2 H), 3.61–3.52 (m, 1H), 3.52–3.41 (m, 2H), 3.30 (s, 3H), 3.02–2.92 (m, 2H), 1.40–1.31 (m, 2H), 1.30–1.17 (m, 8H), 0.90–0.82 (m, 3H); ^13^C NMR (100 MHz, DMSO) δ 159.5, 157.9, 130.0, 127.7, 113.2, 100.8, 99.5, 81.9, 68.5, 68.0, 62.5, 55.0, 54.7, 54.6, 39.2, 31.0, 30.0, 29.9, 26.3, 22.0, 13.9; HRMS: [M + H]^+^ calcd for C_23_H_37_N_2_O_7_, 453.2601; found, 453.2594.

## Supporting Information

File 1 Additional experimental data.
