# Supplementary material for: Synthesis and characterization of pH responsive D-glucosamine based molecular gelators
Source: Beilstein J Org Chem. 2014 Dec 23;10:3111–21. doi: 10.3762/bjoc.10.328 (PMC4311663; doi:10.3762/bjoc.10.328)
Supplement: File 1 — Additional experimental data. [file Beilstein_J_Org_Chem-10-3111-s001.pdf]

Supporting Information

for

Synthesis and characterization of pH responsive D-glucosamine based molecular gelators

Navneet Goyal, Hari P. R. Mangunuru, Bargav Parikh, Sonu Shrestha, Guijun Wang*[§]

Address: Department of Chemistry and Biochemistry, Old Dominion University, 4541 Hampton Boulevard, Norfolk, VA 23529, USA

Email: Wang Guijun - g1wang@odu.edu

*Corresponding author

Table of contents……………………………………………………………………………...S1

Figure S1………………………………………………………………………………….…...S2

Figure S2………………………………………………………………………………….…...S3

Figure S3………………………………………………………………………………….…...S4

Figure S4………………………………………………………………………………….…...S5

The characterization data for compounds **10–14**, **17–22**……………………………………S6

Synthesis of compound **3** …………………………………………………………………S10

^{1}H and ^{13}C NMR spectra of compounds **7–22**………………………………………….S11

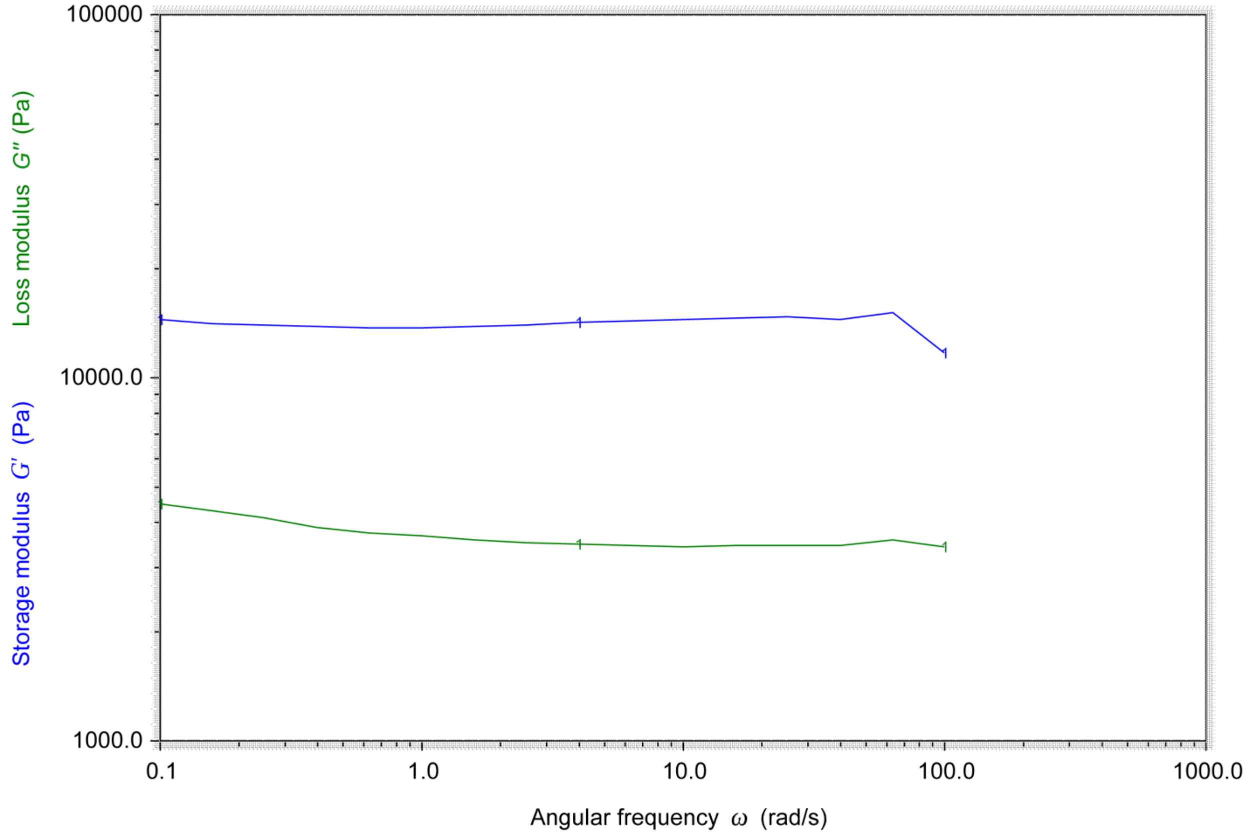

Figure S1: Rheology property of compound **17** in DMSO/H_2O, 1:2, 4.0 mg/mL.

Figure S2: Gel stability under neutral conditions.

Compound	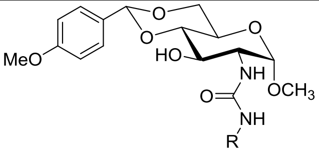 R =	pH	DMSO/ H_2O (1:2)	After 2 h and 20 h
**16**	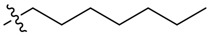	7	G 2.0	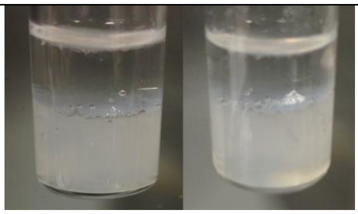
**17**	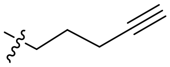	7	G 4.0	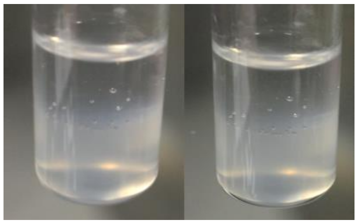
**20**	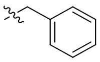	7	G 4.0	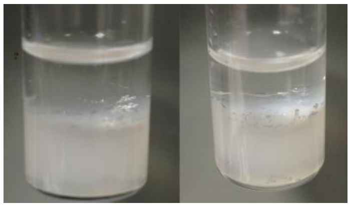
**21**	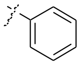	7	G 3.0	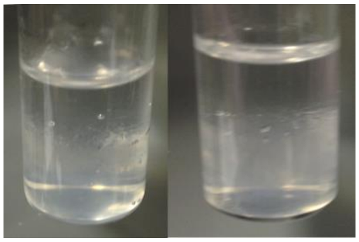

To 1 mL of DMSO/H_2O gels at their MGCs in a vial, 1 mL of water (pH 7) was added, a photo of the gel was taken at 2 h and 20 h, there is almost no change of the gel integrity.

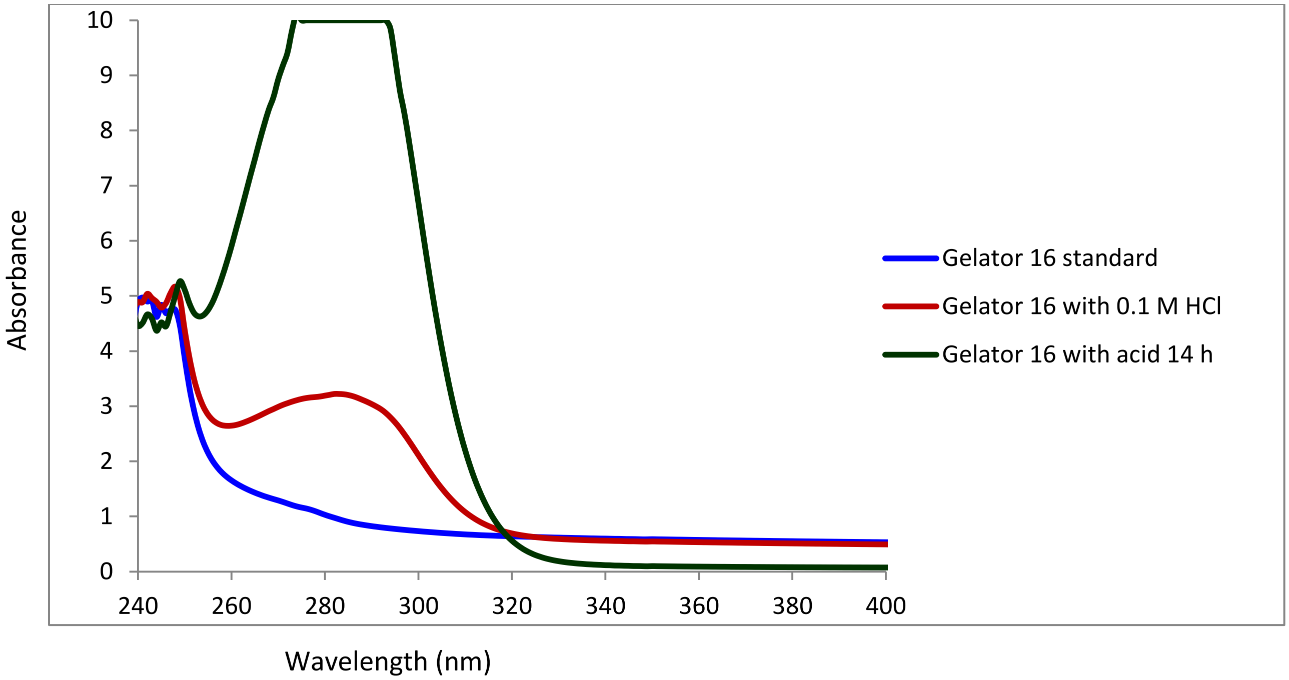

Figure S3: UV spectra of the gelator compound **16** in the same experimental conditions. The blue line is corresponding to the gelator **16**, 6 mg in 3 mL of DMSO/H_2O (1:2) and 3 mL of water. The red line is corresponding to the gelator **16**, 6 mg in 3 mL of DMSO/H_2O (1:2) and 3 mL of water after 10 minutes of addition of 0.1 M HCl acid. The dark green line is the same sample as the red curve but after 16 hours. The absorbance at λ_{max} 285 nm is due to the *p*-methoxybenzaldehyde.

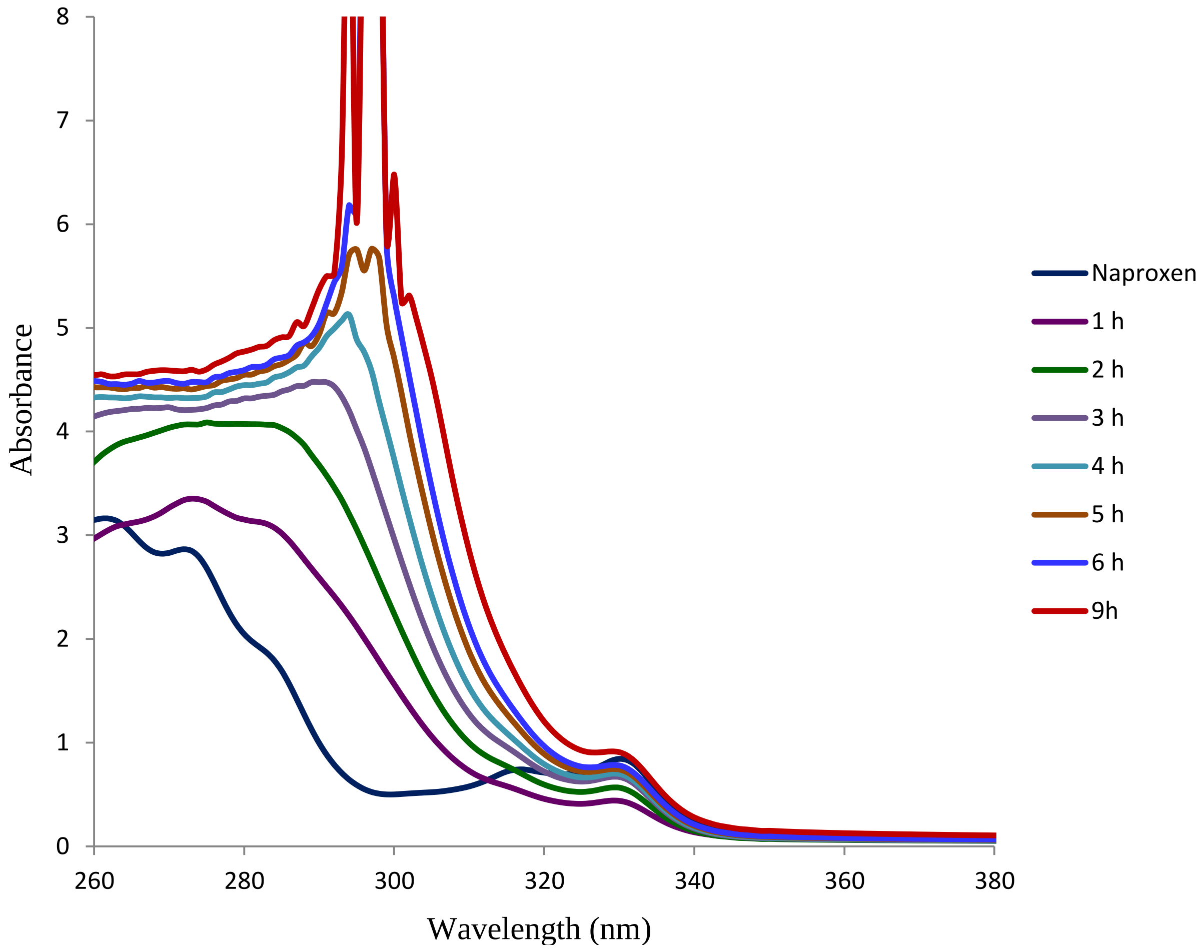

Figure S4: Naproxen release study from the gel formed by compound **16** in presence of 0.1 M of HCl solution. For clarity, the spectra are only showing the absorptions for naproxen.

The characterization data for compounds **10–14**, and **17–22:**

Compound 10, octylamide, isolated as a white solid in 82% yield, mp 174.2 – 175.0 ^{o} C. ^{1}H NMR (400 MHz, $CDCl_3$) δ 7.38-7.44 (m, 2H), 6.83-6.91 (m, 2H), 5.90 (d, 1H, *J* = 8.4 Hz), 5.51 (s, 1H), 4.70 (d, 1H, *J* = 3.7 Hz), 4.16-4.28 (m, 2H), 3.82-3.91 (m, 1H), 3.78 (s, 3H), 3.68-3.77 (m, 2H), 3.50-3.59 (m, 1H), 3.38 (s, 3H), 2.22 (t, 2H, *J* = 7.3 Hz), 1.56-1.68 (m, 2H), 1.18-1.35 (m, 8H), 0.87 (t, 3H, *J* = 7.0 Hz). ^{13}C NMR (100 MHz, $CDCl_3$) δ 174.6, 160.1, 129.6, 127.6, 113.5, 101.8, 98.8, 82.0, 70.5, 68.7, 62.3, 55.2, 53.9, 36.6, 31.6, 29.0, 28.9, 25.5, 22.5, 14.0. HRMS calcd for $C_{23}H_{36}NO_7$ $[M+H]^+$438.2492, found 438.2493.

Compound 11, 5-hexynoic amide, isolated as a white solid in 87% yield, mp 204.0 – 205.0 ^{o} C. ^{1}H NMR (400 MHz, $CDCl_3$) δ 7.38-7.44 (m, 2H), 6.85-6.91 (m, 2H), 5.89 (d, 1H, *J* = 8.4 Hz), 5.51 (s, 1H), 4.71 (d, 1H, *J* = 3.7 Hz), 4.24-4.29 (m, 1H), 4.22 (dd, 1H, J = 3.7, 9.9 Hz), 3.86-3.94 (m, 1H), 3.80 (s, 3H), 3.72-3.79 (m, 2H), 3.53-3.60 (m, 1H), 3.41(s, 3H), 2.98 (d, 1H, *J* = 3.3 Hz), 2.40 (t, 2H, *J* = 7.3 Hz), 2.25-2.32 (m, 2H), 1.99 (t, 1H, *J* = 2.6 Hz), 1.82-1.94 (m, 2H). ^{13}C NMR (100 MHz, $CDCl_3$) δ 173.6, 160.2, 129.6, 127.6, 113.7, 101.9, 98.8, 83.4, 82.0, 75.6, 70.9, 69.3, 68.8, 62.3, 55.3, 53.9, 34.9, 23.9, 17.6. HRMS calcd for $C_{21}H_{28}NO_7$ $[M+H]^+$406.1866, found 406.1865.

Compound 12, 5-hexenylamide, isolated as a white solid in 62 % yield, mp 158.4 – 159.2 ^{o} C ^{1}H NMR (400 MHz, $CDCl_3$) δ 7.38-7.44 (m, 2H), 6.84-6.91 (m, 2H), 5.86 (d, 1H, *J* = 8.4 Hz), 5.70-5.83 (m, 1H), 5.51 (s, 1H), 4.95-5.07 (m, 2H), 4.71 (d, 1H, *J* = 3.7 Hz), 4.16-4.30 (m, 2H), 3.82-3.94 (m, 1H), 3.79 (s, 3H), 3.70-3.78 (m, 2H), 3.51-3.60 (m, 1H), 3.39 (s, 3H), 3.18 (sb, 1H), 2.24 (t, 2H, *J* = 7.3 Hz), 2.04-2.15 (m, 2H), 1.68-1.82 (m, 2H). ^{13}C NMR (100 MHz,

$CDCl_3$) δ 174.3, 160.2, 137.8, 129.6, 127.6, 115.4, 113.6, 101.8, 98.8, 82.0, 70.7, 68.7, 62.3, 55.3, 53.9, 35.7, 32.9, 24.5. HRMS calcd for $C_{21}H_{30}NO_7$ $[M+H]^+$408.2022, found 408.2018.

Compound 13, phenylamide, isolated as a white solid in 84% yield, mp 243.2 – 244.2 °C. ^{1}H NMR (400 MHz, $CDCl_3$) δ 7.78-7.83 (m, 2H), 7.49-7.55 (m, 1H), 7.40-7.47 (m, 4H), 6.86-6.92 (m, 2H), 6.54 (d, 1H, *J* = 8.4 Hz), 5.51 (s, 1H), 4.84 (d, 1H, *J* = 3.7 Hz), 4.40-4.48 (m, 1H), 4.28 (dd, 1H, *J* = 3.7, 9.2 Hz), 3.96-4.06 (m, 1H), 3.73-3.81 (m, 2H), 3.79 (s, 3H), 3.59-3.66 (m, 1H), 3.43 (s, 3H), 3.19 (d, 1H, *J* = 3.3 Hz). ^{13}C NMR (100 MHz, $CDCl_3$) δ 168.4, 160.2, 133.6, 131.9, 129.6, 128.6, 127.6, 127.2, 115.3, 113.6, 101.9, 98.9, 82.0, 70.7, 68.8, 62.4, 55.3, 55.26, 54.4. HRMS calcd for $C_{22}H_{26}NO_7$ $[M+H]^+$416.1709, found 416.1704.

Compound 14, napthylamide, isolated as a white solid in 83% yield, mp 242.5–244.0 °C. ^{1}H NMR (400 MHz, $CDCl_3$) δ 8.35 (d, 1H, *J* = 7.7 Hz), 7.95 (d, 1H, *J* = 8.1 Hz), 7.86-7.90 (m, 1H), 7.67 (d, 1H, *J* = 7.0 Hz), 7.51-7.59 (m, 2H), 7.46-7.51 (m, 1H), 7.41-7.46 (m, 2H), 6.87-6.92 (m, 2H), 6.36 (d, 1H, *J* = 8.4 Hz), 5.57 (s, 1H), 4.93 (d, 1H, *J* = 3.7 Hz), 4.56 (dt,1H, *J* = 3.7, 9.5 Hz), 4.28-4.33 (m, 1H), 4.01-4.09 (m, 1H), 3.81 (s, 3H), 3.77-3.87 (m, 2H), 3.63-3.70 (m, 1H), 3.43 (s, 3H), 3.03-3.08 (m, 1H). ^{13}C NMR (100 MHz, $CDCl_3$) δ 170.6, 160.1, 133.7, 133.5, 130.8, 129.9, 129.5, 128.2, 127.5, 127.1, 126.4, 125.2, 125.1, 124.6, 113.5, 101.8, 98.9, 82.0, 69.7, 68.7, 62.6, 55.3, 55.2, 54.3. HRMS calcd for $C_{26}H_{28}NO_7$ $[M+H]^+$466.1866, found 466.1864.

Compound 17, 4-pentynylurea, isolated as a white solid in 87% yield, mp 203.2–204.2 °C. ^{1}H NMR (400 MHz, DMSO) δ (ppm) 7.33-7.39 (m, 2H), 6.88-6.94 (m, 2H), 6.12 (t, 1H, *J* = 5.5

Hz), 5.78 (d, 1H, *J* = 8.4 Hz), 5.54 (s, 1H), 5.18 (d, 1H, *J* = 5.5 Hz), 4.61 (d, 1H, *J* = 3.7 Hz), 4.14 (dd, 1H, *J* = 4.4, 9.9 Hz), 3.75 (s, 3H), 3.63-3.73 (m, 2 H), 3.52-3.61 (m, 1H), 3.40-3.52 (m, 2H), 3.29 (s, 3H), 3.05 (q, 2H, *J* = 6.6 Hz), 2.75-2.81 (m, 1H), 2.10-2.20 (m, 2H), 1.53 (p, 2H, *J* = 7.0 Hz). ^{13}C NMR (100 MHz, DMSO) δ 159.5, 157.9, 130.1, 127.7, 113.2, 100.8, 99.4, 84.0, 81.9, 71.3, 68.5, 68.0, 62.5, 55.0, 54.7, 54.5, 38.2, 28.8, 15.2. HRMS calcd for $C_{21}H_{29}N_2O_7$ $[M+H]^+$421.1975, found 421.1978.

Compound 18, 5-hexynylurea, isolated as a yellow solid in 92% yield, mp 174.2-175.0 ^{o}C ^{1}H NMR (400 MHz, DMSO) δ (ppm) 7.31-7.39 (m, 2H), 6.88-6.98 (m, 2H), 6.08 (t, 1H, *J* = 5.5 Hz), 5.78 (d, 1H, *J* = 8.4 Hz), 5.53 (s, 1H), 5.19 (d, 1H, *J* = 5.5 Hz), 4.61 (d, 1H, *J* = 3.7 Hz), 4.14 (dd, 1H, *J* = 4.8, 9.9 Hz), 3.75 (s, 3H), 3.64-3.73 (m, 2 H), 3.53-3.61 (m, 1H), 3.41-3.53 (m, 2H), 3.30 (s, 3H), 2.94-3.05 (m, 2H), 2.74 (t, 1H, *J* = 2.6 Hz), 2.09-2.21 (m, 2H), 1.36-1.50 (m, 4H). ^{13}C NMR (100 MHz, DMSO) δ 159.5, 158.0, 130.1, 127.7, 113.3, 100.8, 99.5, 84.4, 82.0, 71.2, 68.5, 68.0, 62.5, 55.1, 54.7, 54.6, 38.6, 29.1, 25.4, 17.4. HRMS calcd for $C_{22}H_{31}N_2O_7$ $[M+H]^+$473.2144, found 473.2136.

Compound 19, cyclohexylurea, isolated as a white solid in quantitative yield, mp 223.4 – 223.9 ^{o}C ^{1}H NMR (400 MHz, DMSO) δ (ppm) 7.33-7.39 (m, 2H), 6.88-6.95 (m, 2H), 6.03 (d, 1H, *J* = 7.7 Hz), 5.72 (d, 1H, *J* = 8.4 Hz), 5.53 (s, 1H), 5.20 (d, 1H, *J* = 5.5 Hz), 4.61 (d, 1H, *J* = 3.7 Hz), 4.14 (dd, 1H, *J* = 4.8, 9.9 Hz), 3.75 (s, 3H), 3.63-3.73 (m, 2 H), 3.51-3.60 (m, 1H), 3.39-3.52 (m, 3H), 3.29 (s, 3H), 1.69-1.78 (m, 2H), 1.57-1.67 (m, 2H), 1.44-1.55 (m, 1H), 1.00-1.32 (m, 5H). ^{13}C NMR (100 MHz, DMSO) δ 159.5, 157.3, 130.1, 127.7, 113.3, 100.8, 99.4, 81.9, 68.6, 68.0,

62.5, 55.0, 54.7, 54.5, 47.6, 33.2, 25.3, 24.3. HRMS calcd for $C_{22}H_{33}N_2O_7$ $[M+H]^+$437.2288, found 437.2291.

Compound 20, benzylurea, isolated as a white solid in quantitative yield, mp 202.2 – 203.5 °C ^1H NMR (400 MHz, DMSO) δ (ppm) 7.34-7.39 (m, 2H), 7.28-7.34 (m, 2H), 7.19-7.27 (m, 3H), 6.88-6.95 (m, 2H), 6.52 (t, 1H, *J* = 5.5 Hz), 5.93 (d, 1H, *J* = 8.4 Hz), 5.54 (s, 1H), 5.21 (d, 1H, *J* = 5.5 Hz), 4.64 (d, 1H, *J* = 3.3 Hz), 4.18-4.24 (m, 2H), 4.10-4.17 (m, 1H), 3.75 (s, 3H), 3.66-3.74 (m, 2 H), 3.42-3.62 (m, 3H), 3.31 (s, 3H). ^{13}C NMR (100 MHz, DMSO) δ 159.5, 157.9, 140.6, 130.1, 128.2, 127.7, 127.0, 126.5, 113.2, 100.7, 99.4, 81.9, 68.4, 68.0, 62.5, 55.0, 54.8, 54.7, 42.8. HRMS calcd for $C_{23}H_{29}N_2O_7$ $[M+H]^+$445.1975, found 445.1975.

Compound 21, phenylurea, isolated as a white solid in 85% yield, mp 236.2 – 237.0 °C ^1H NMR (400 MHz, DMSO) δ (ppm) 8.62 (s, 1H), 7.34-7.45 (m, 4H), 7.18-7.26 (m, 2H), 6.86-6.95 (m, 3H), 6.13 (d, 1H, *J* = 8.8 Hz), 5.56 (s, 1H), 5.31 (d, 1H, *J* = 5.9 Hz), 4.70 (d, 1H, *J* = 3.7 Hz), 4.14 (dd, 1H, *J* = 4.7, 9.9 Hz), 3.75 (s, 3H), 3.70-3.83 (m, 2H), 3.46-3.66 (m, 3H), 3.33 (s, 3H) ^{13}C NMR (100 MHz, DMSO) δ 159.5, 154.9, 140.3, 130.1, 128.7, 127.7, 121.1, 117.4, 113.3, 100.8, 99.3, 81.8, 68.4, 68.0, 62.6, 55.1, 54.7, 54.2. HRMS calcd for $C_{22}H_{27}N_2O_7$ $[M+H]^+$431.1818, found 431.1814.

Compound 22, naphthylurea, isolated as a yellow solid in quantitative yield, mp 263.0-264.0 °C, ^1H NMR (400 MHz, DMSO) δ (ppm) 8.72 (s, 1H), 8.14 (d, 1H, *J* = 8.1 Hz), 8.09 (d, 1H, *J* = 7.7 Hz), 7.89 (d, 1H, *J* = 7.7 Hz), 7.48-7.60 (m, 3H), 7.33-7.46 (m, 3H), 6.89-6.98 (m, 2H), 6.79 (d,

1H, *J* = 8.8 Hz), 5.58 (s, 1H), 5.40 (d, 1H, *J* = 4.8 Hz), 4.64 (d, 1H, *J* = 3.7 Hz), 4.19 (dd, 1H, *J* = 4.4, 9.9 Hz), 3.83-3.91 (m, 1H), 3.69-3.77 (m, 4H), 3.47-3.68 (m, 3 H), 3.37 (s, 3H). ^{13}C NMR (100 MHz, DMSO) δ 159.6, 155.5, 135.1, 133.8, 130.2, 128.5, 127.8, 126.0, 125.8, 125.5, 125.1, 122.0, 121.2, 115.9, 113.4, 100.9, 99.4, 81.9, 68.6, 68.1, 62.7, 55.2, 54.9, 54.6. HRMS calcd for $C_{26}H_{29}N_2O_7$ $[M+H]^+$481.1975, found 481.1980.

Procedure for the synthesis of 3:

N-Acetyl-D-glucosamine **4** (5.0 g, 21.3 mmol) was added to a flask containing methanol (50.0 mL) and Amberlite IR-120 resin (5.00 g), this reaction mixture was refluxed overnight. After this the resin was removed and washed with methanol, the combined organic phase was concentrated on a rotavap under vacuum, the product **5** (4.9 g, 90%) was obtained as a mixture of anomers (α/β ~8:1). This product was then directly carried to the next step, the crude product **5** was added to 25.0 mL of anhydrous DMF, followed by *p*-methoxydimethylbenzylidene acetal (6.45 g, 35.4 mmol) and p-toluenesulfonic acid (0.3 g, 2.08 mmol). The reaction mixture was heated at 60 °C for 2h. After which the methanol was removed under a rotavap, then DMF was removed in vacuum to obtain compound **6** (5.5 g) as white solid. This crude product was then purified by flash chromatography to yield compound **6**. The α anomer was the major product and this was then treated with 75.0 mL of refluxing 3 N KOH in ethanol for 18 hours. The reaction mixture was diluted with a 2% MeOH in DCM (~150 mL) and water (2 × 100 mL). The organic phase was removed under reduced pressure, and the crude product was purified by column chromatography using 2% MeOH in DCM to give compound **3** in 71% yield.

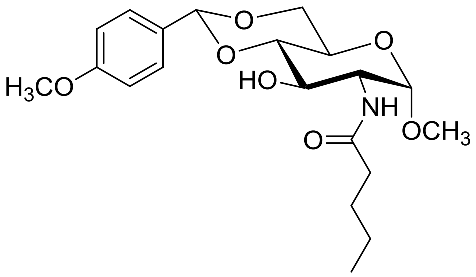

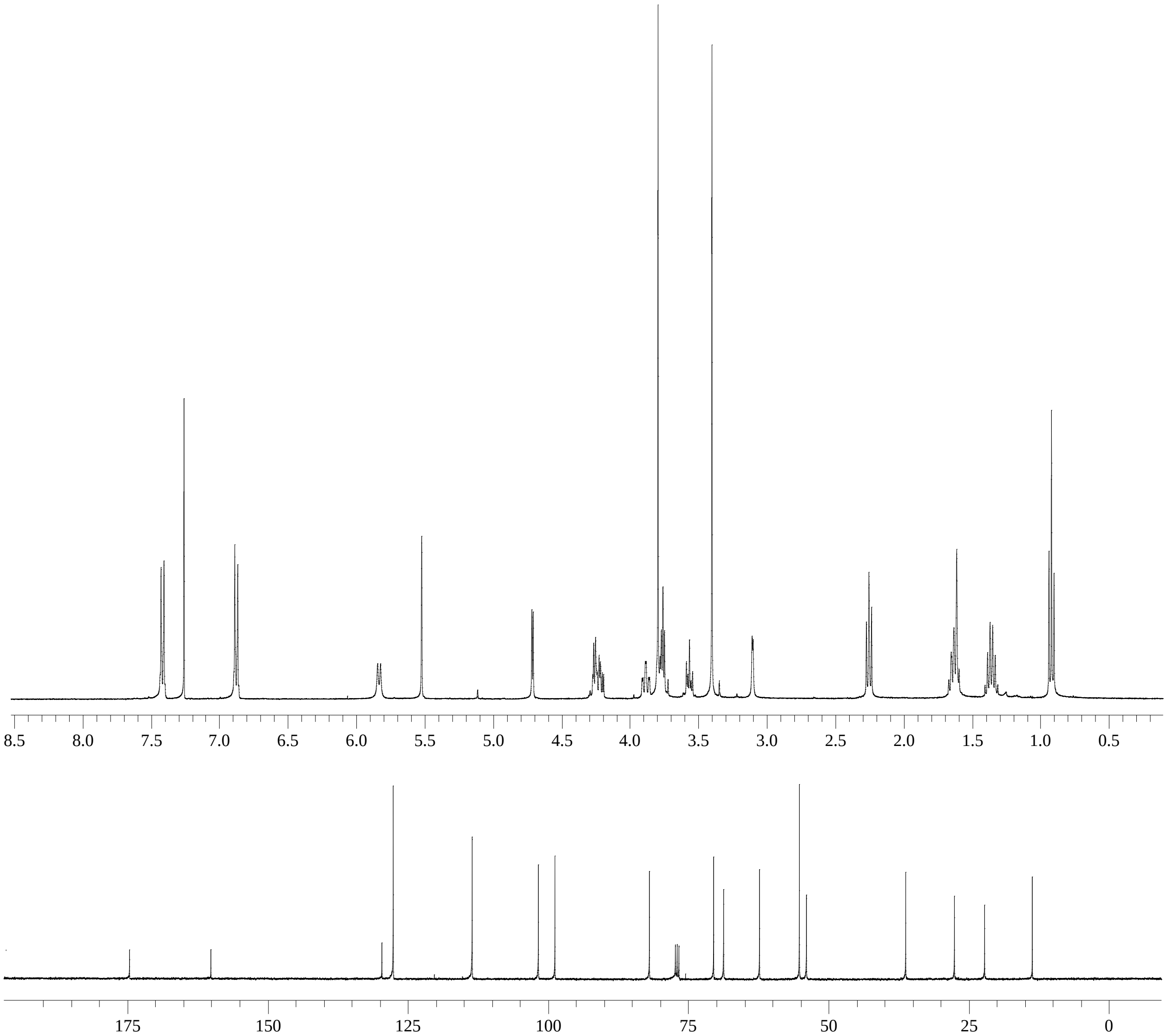

^{1}H and ^{13}C NMR spectra for compound **7**, 400 MHz machine and in $CDCl_3$

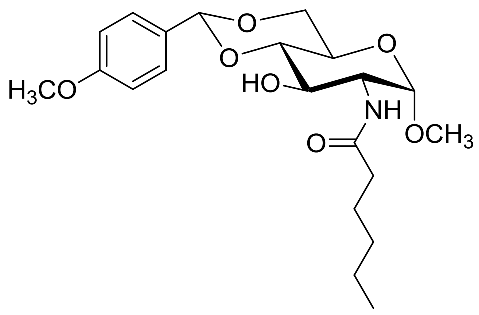

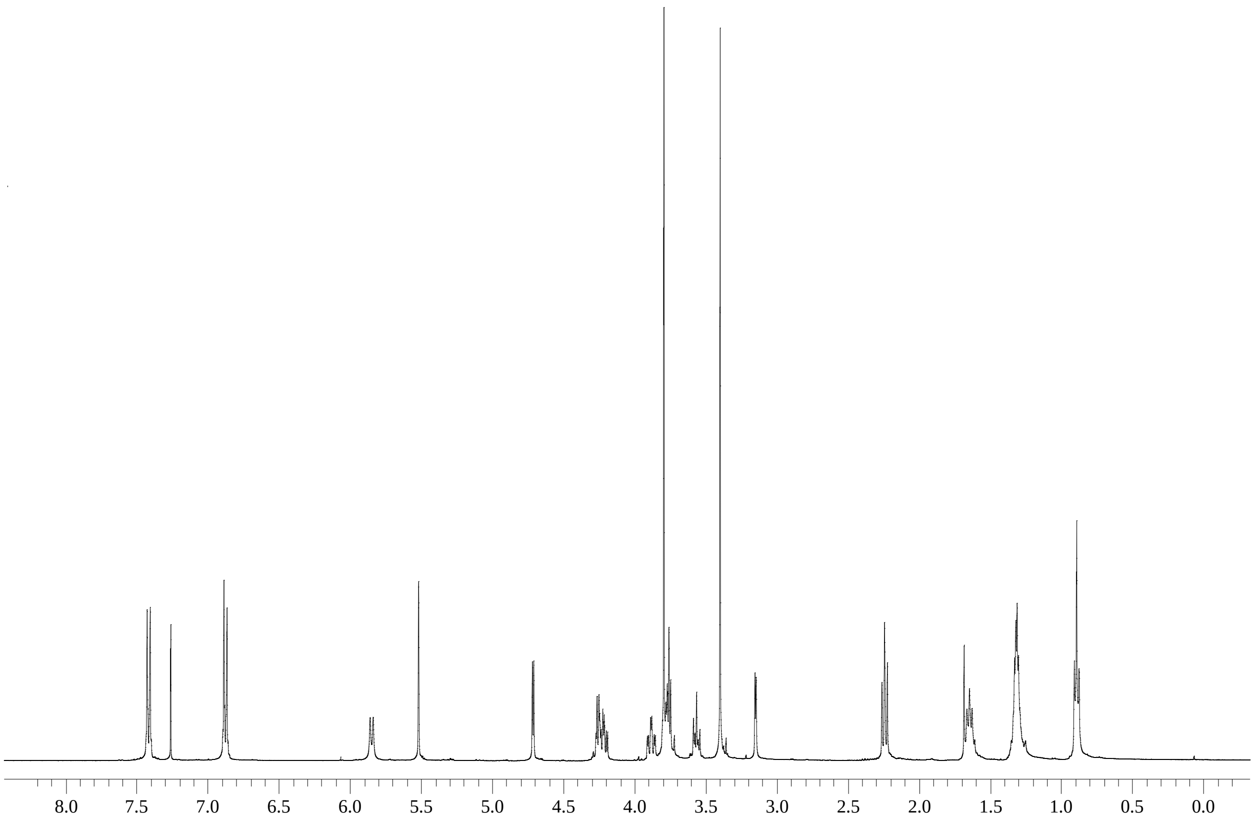

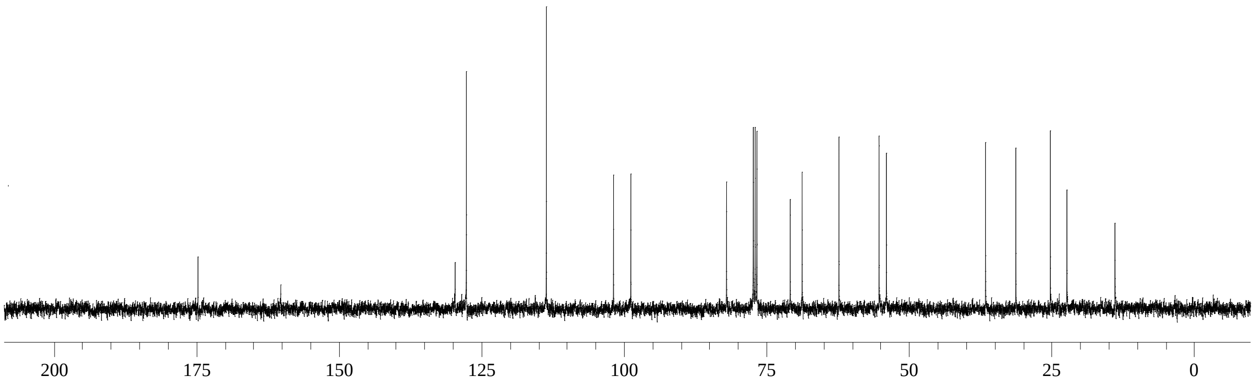

^{1}H and ^{13}C NMR spectra for compound **8**, 400 MHz machine and in $CDCl_3$

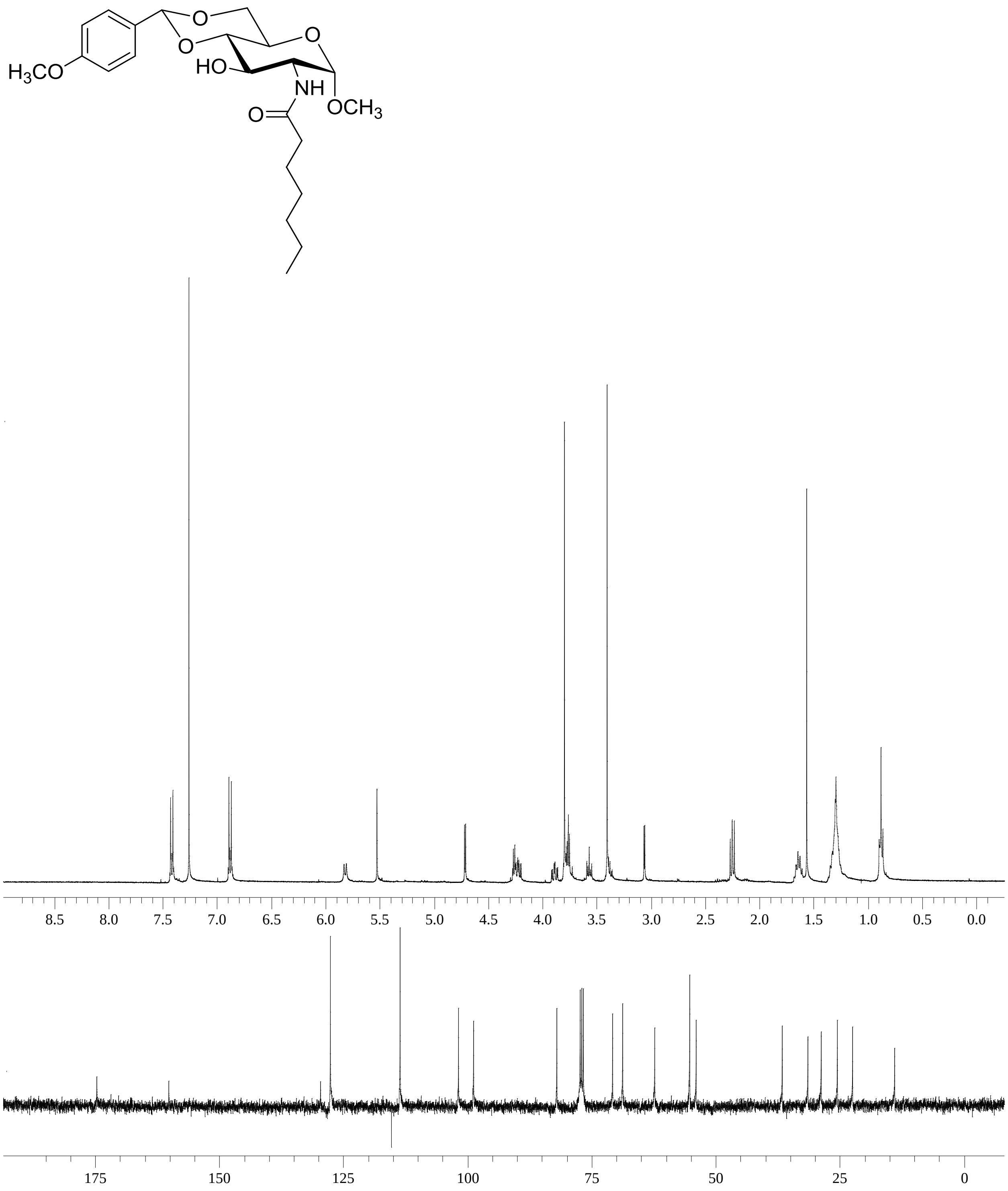

^{1}H and ^{13}C NMR spectra for compound **9**, 400 MHz machine and in $CDCl_3$

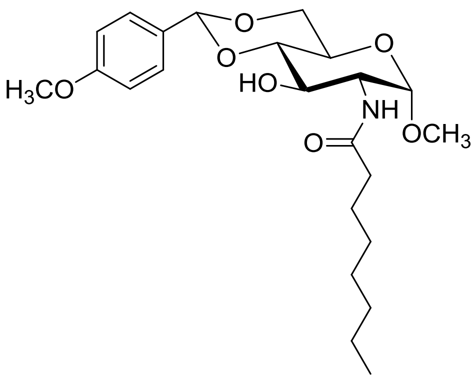

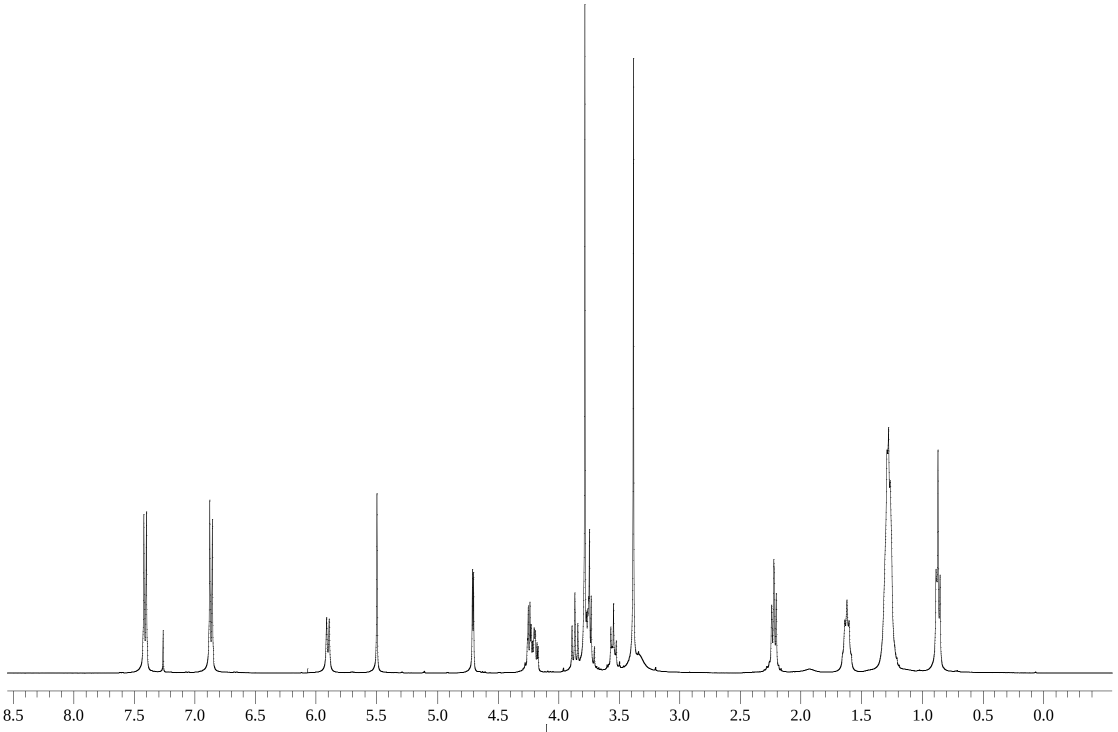

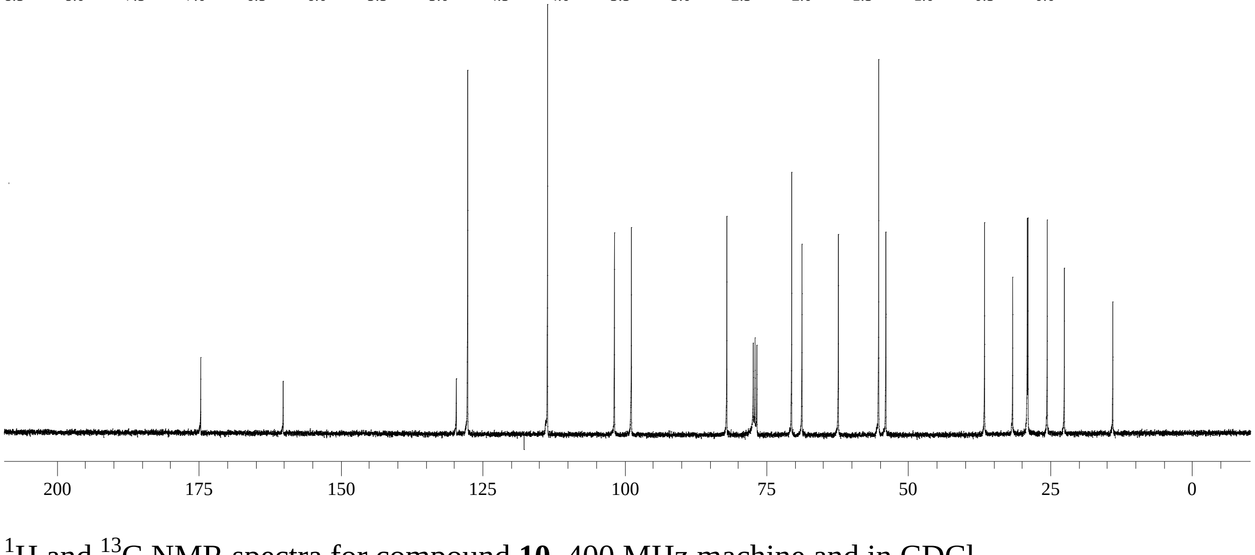

^{1}H and ^{13}C NMR spectra for compound **10**, 400 MHz machine and in $CDCl_3$

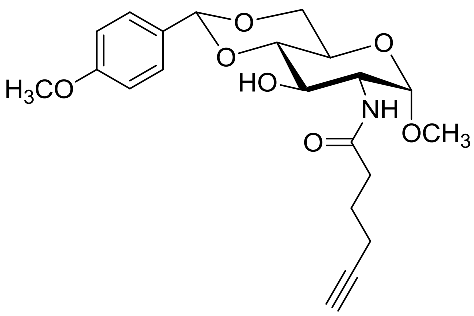

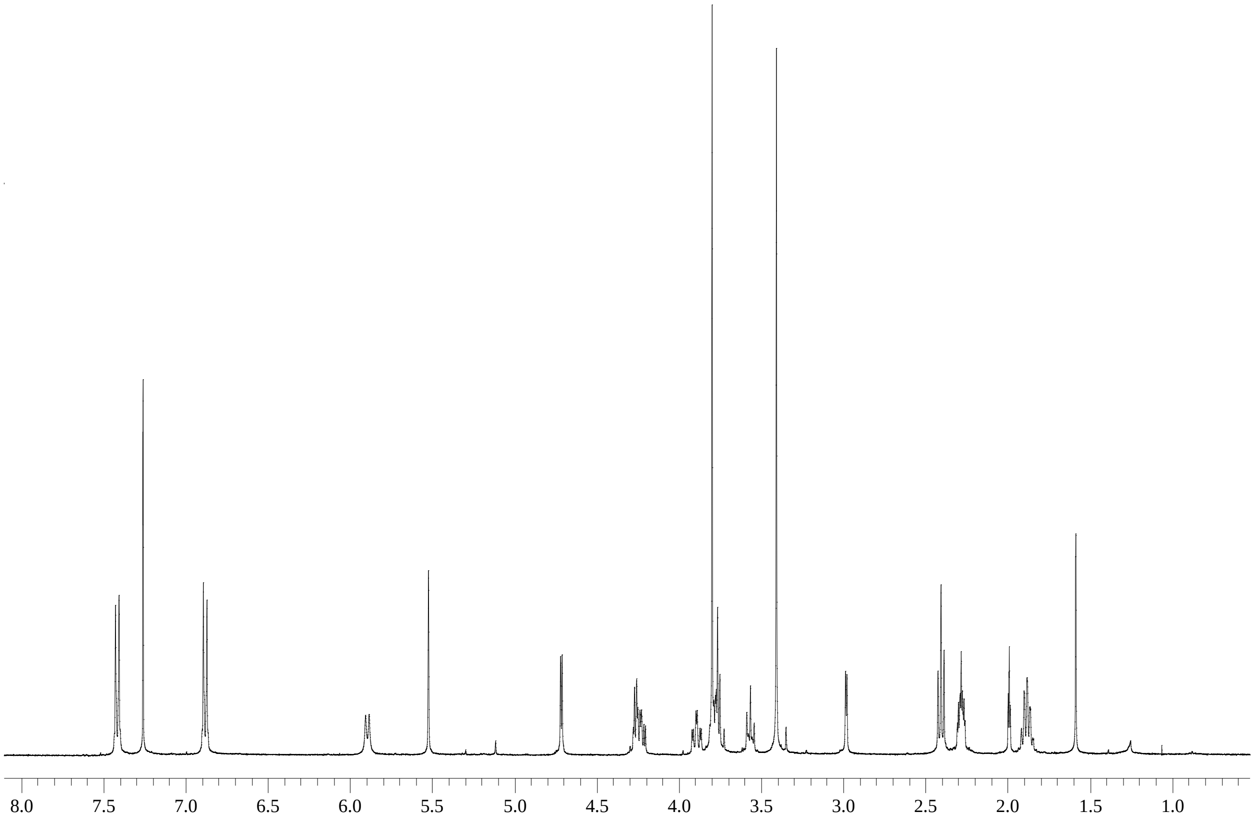

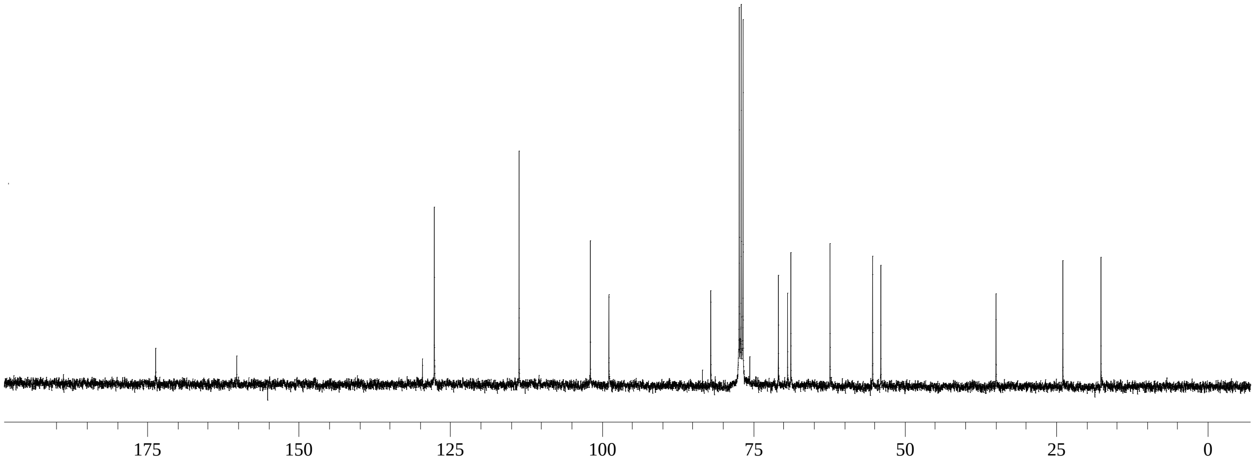

^{1}H and ^{13}C NMR spectra for compound **11**, 400 MHz machine and in $CDCl_3$

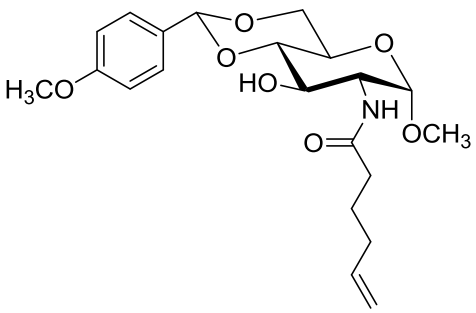

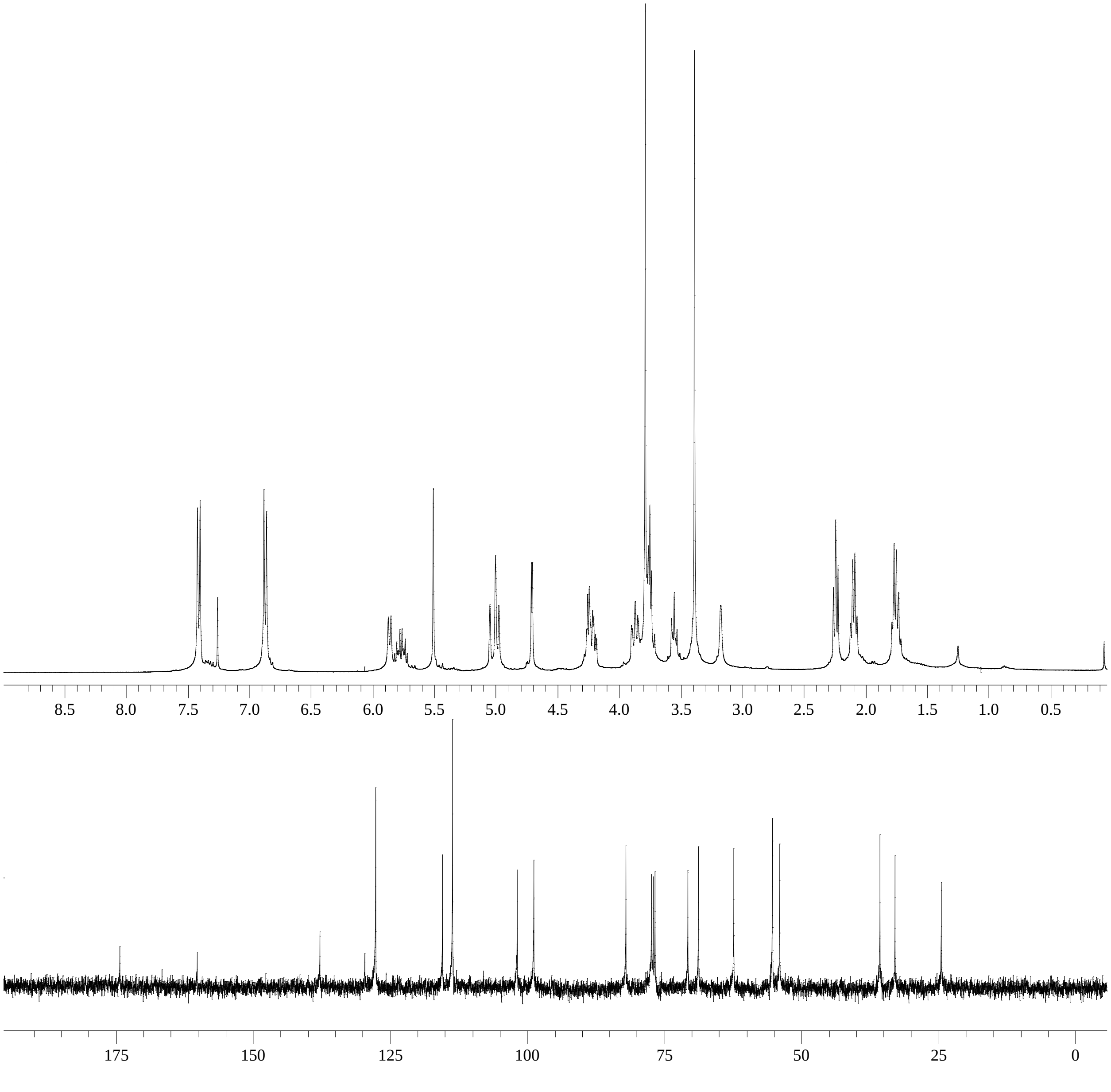

^{1}H and ^{13}C NMR spectra for compound **12**, 400 MHz machine and in $CDCl_3$

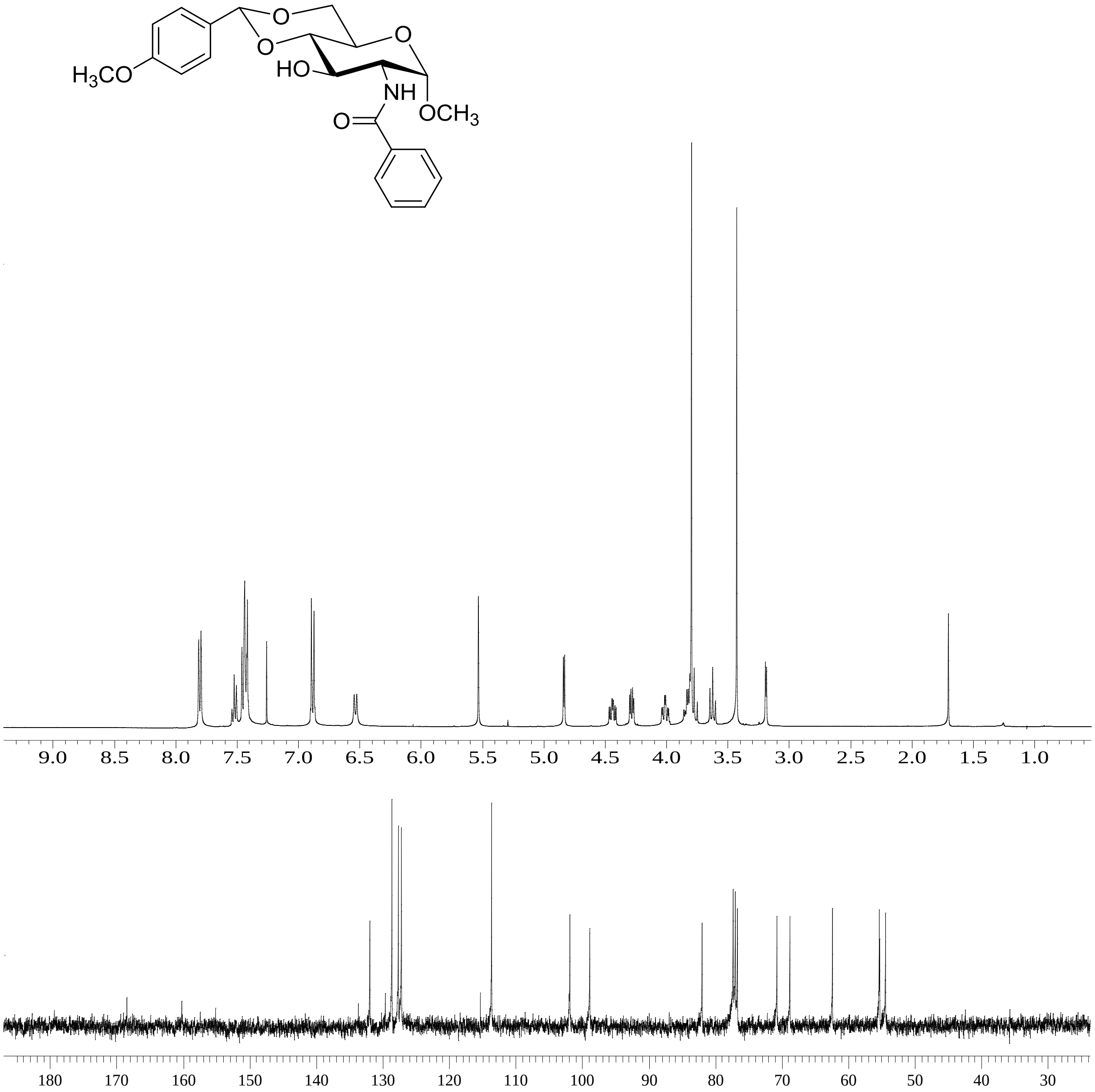

^{1}H and ^{13}C NMR spectra for compound **13**, 400 MHz machine and in $CDCl_3$

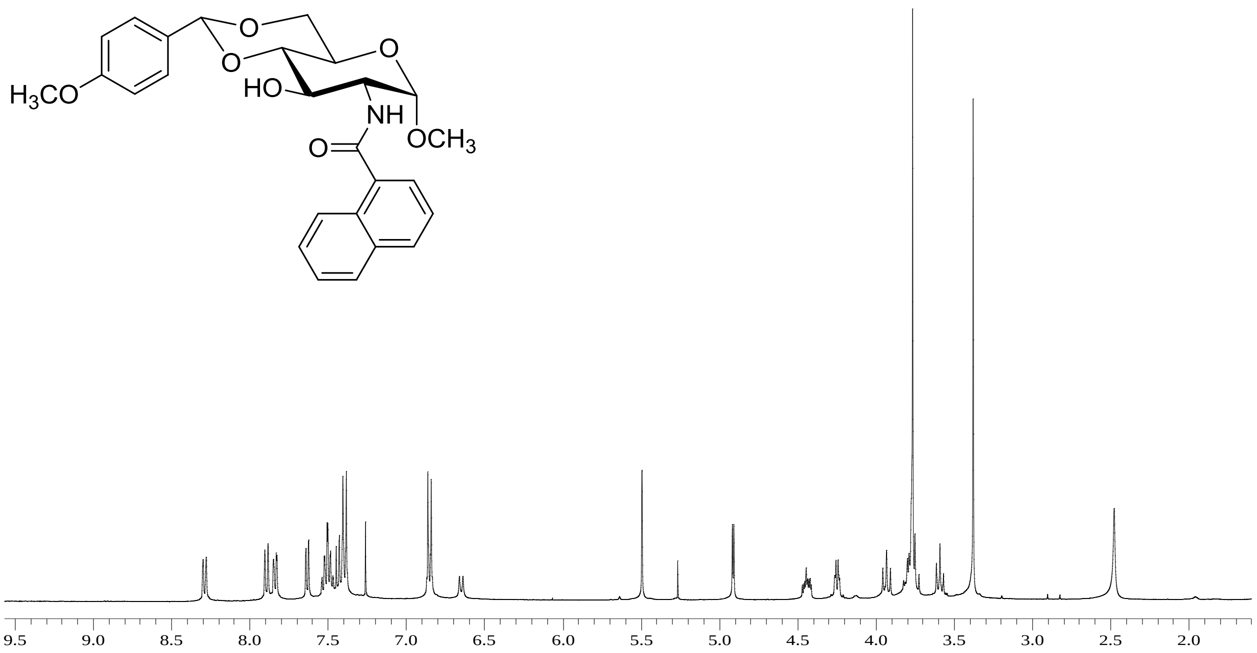

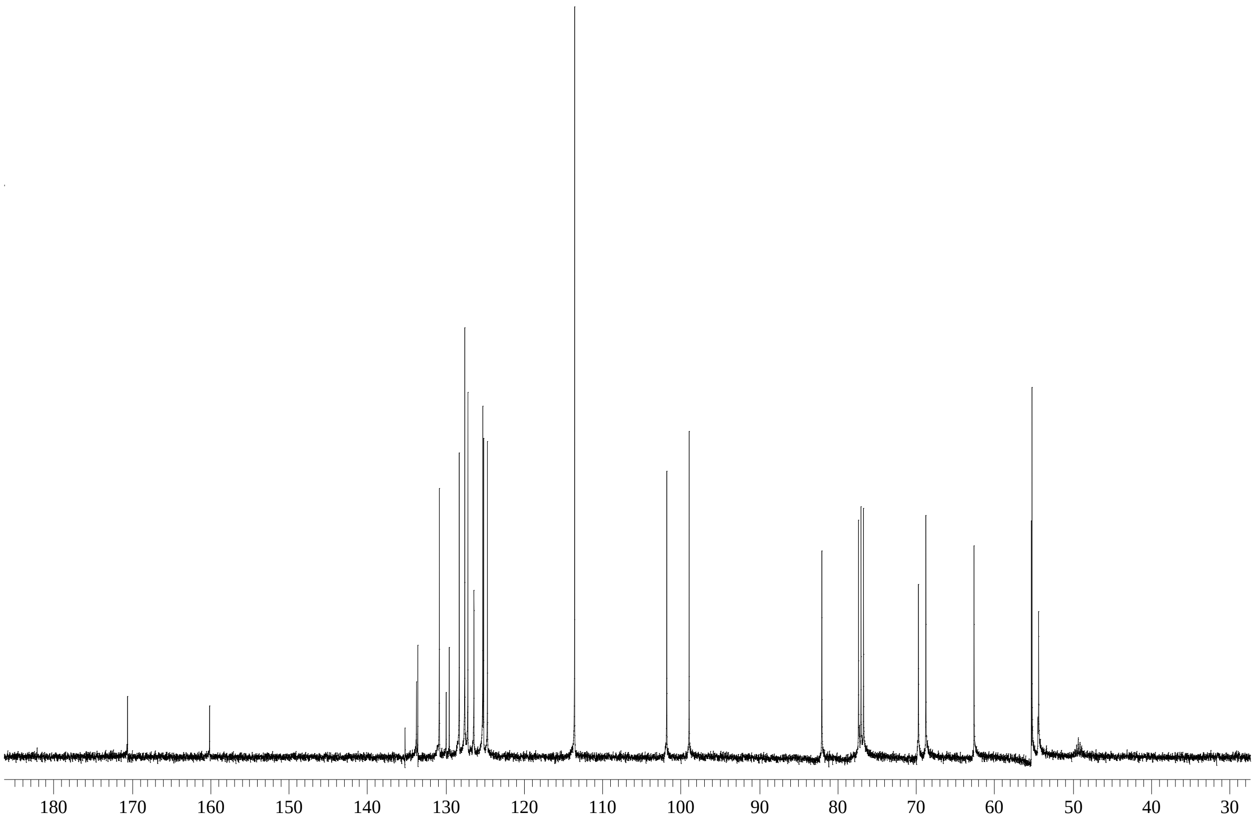

^{1}H and ^{13}C NMR spectra for compound **14**, 400 MHz machine and in $CDCl_3$

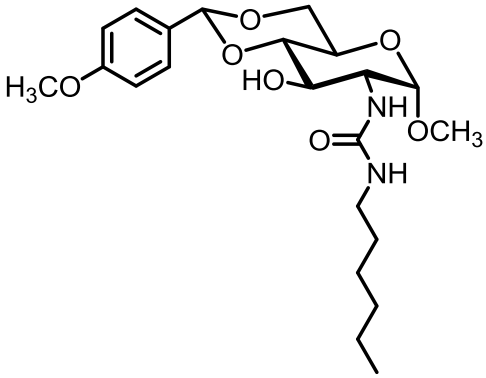

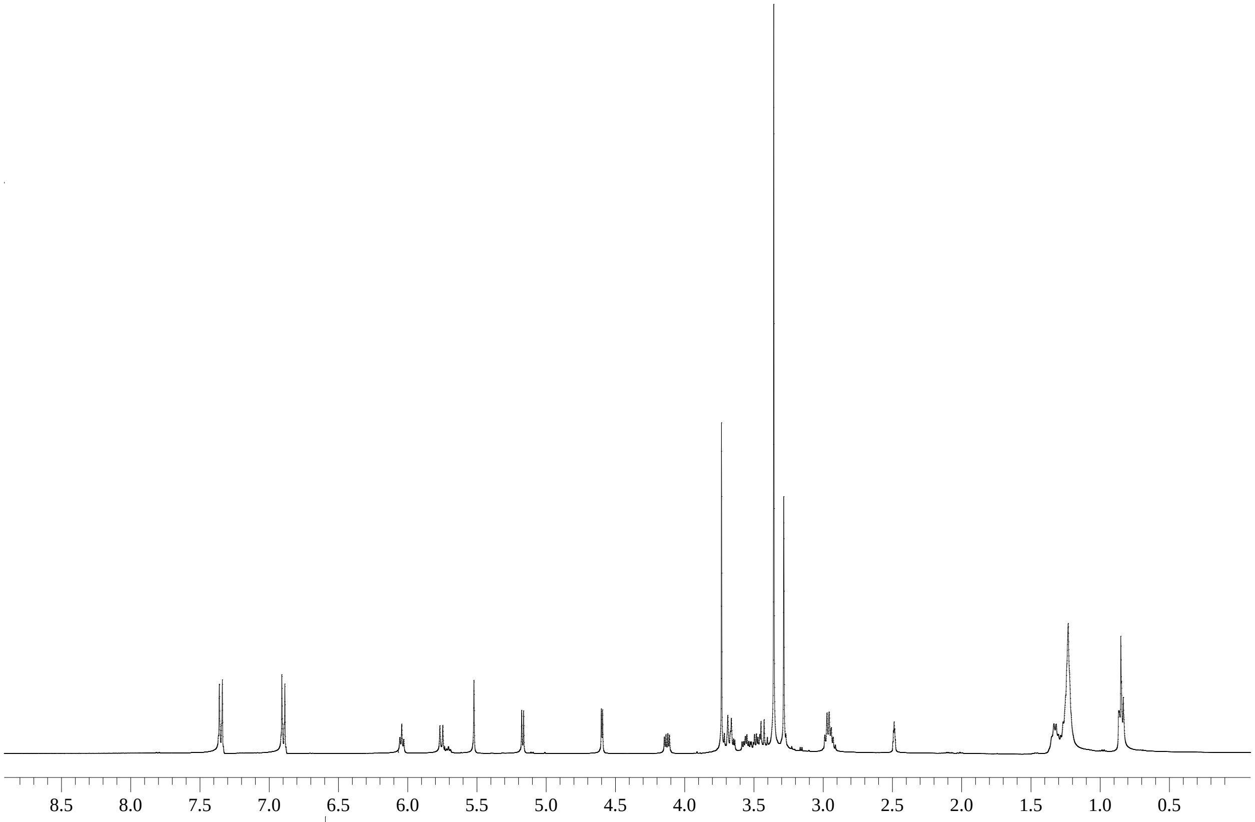

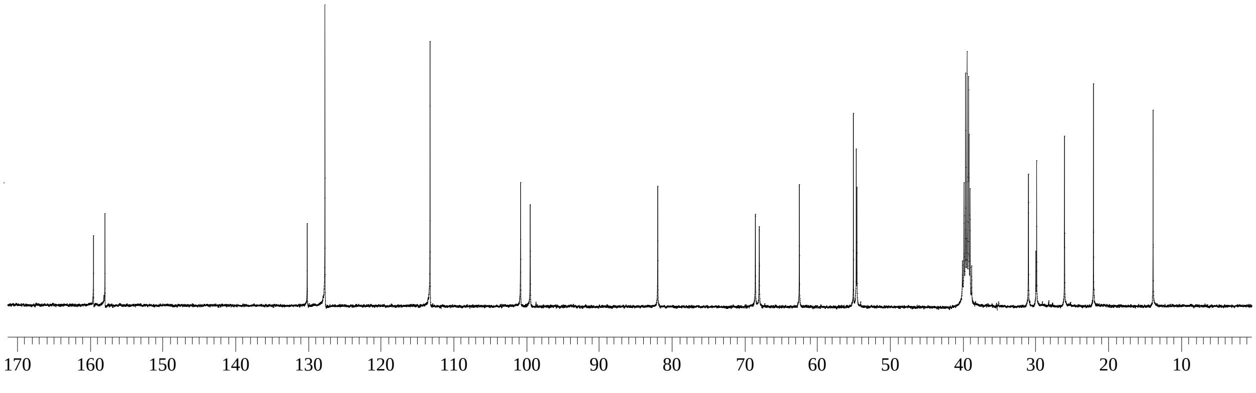

^{1}H and ^{13}C NMR spectra for compound **15**, 400 MHz machine and in DMSO

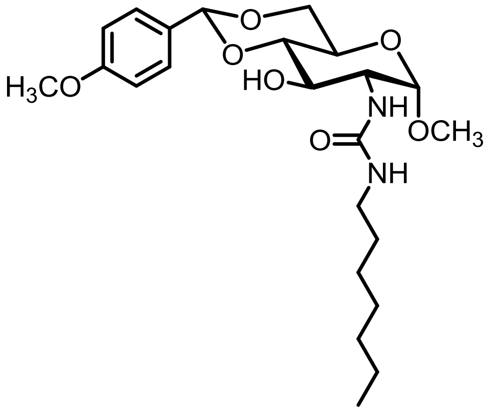

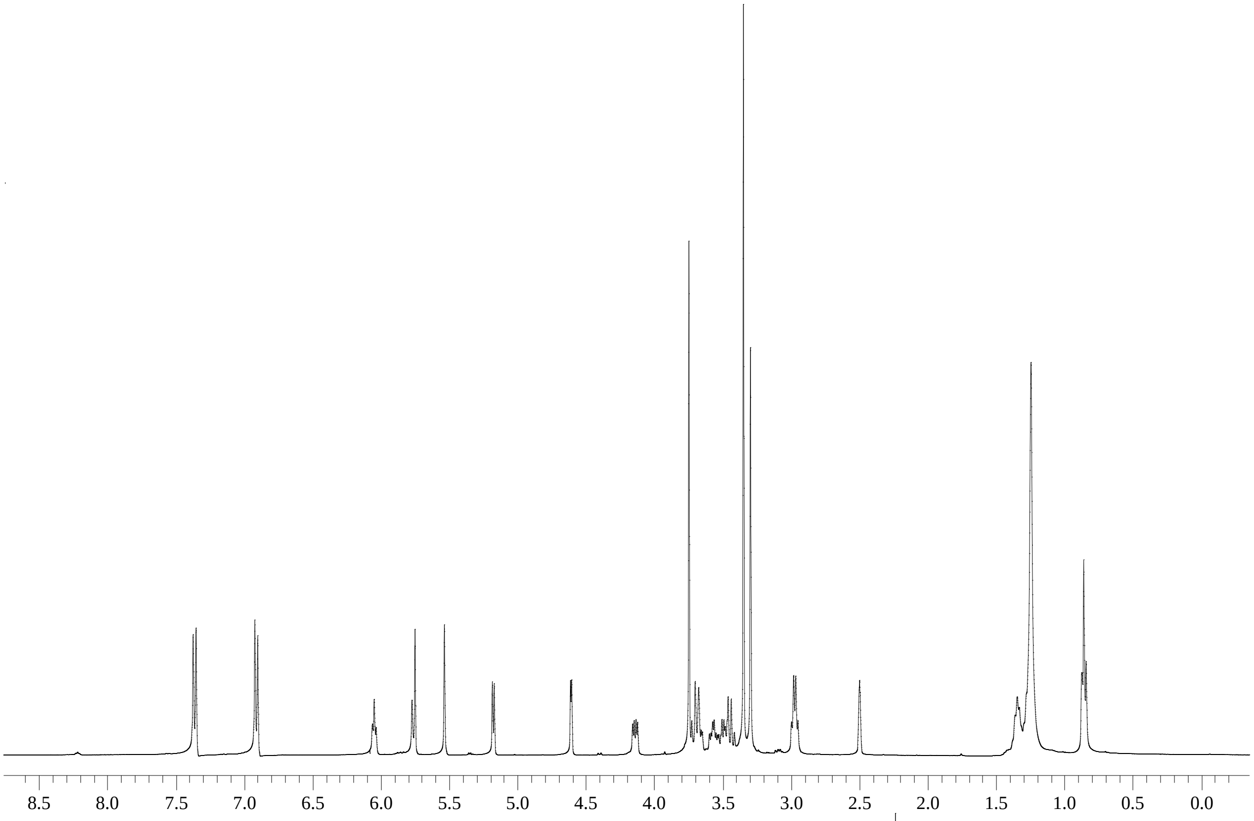

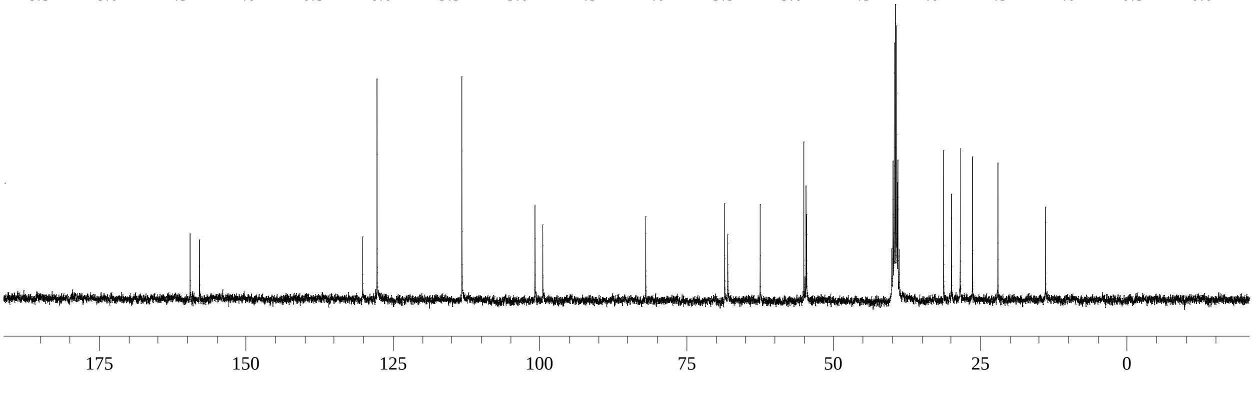

^{1}H and ^{13}C NMR spectra for compound **16**, 400 MHz machine and in DMSO

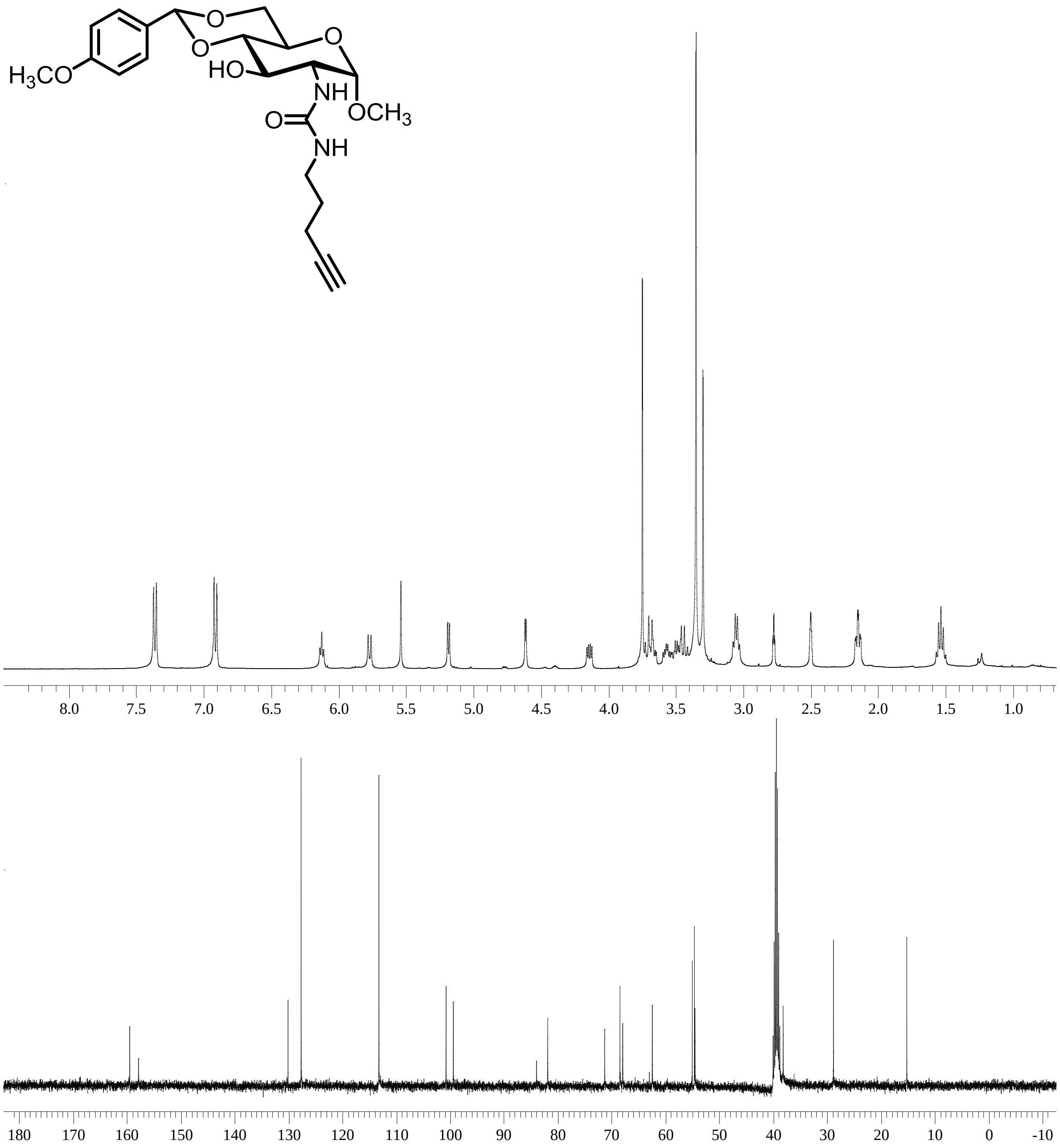

^{1}H and ^{13}C NMR spectra for compound **17**, 400 MHz machine and in DMSO

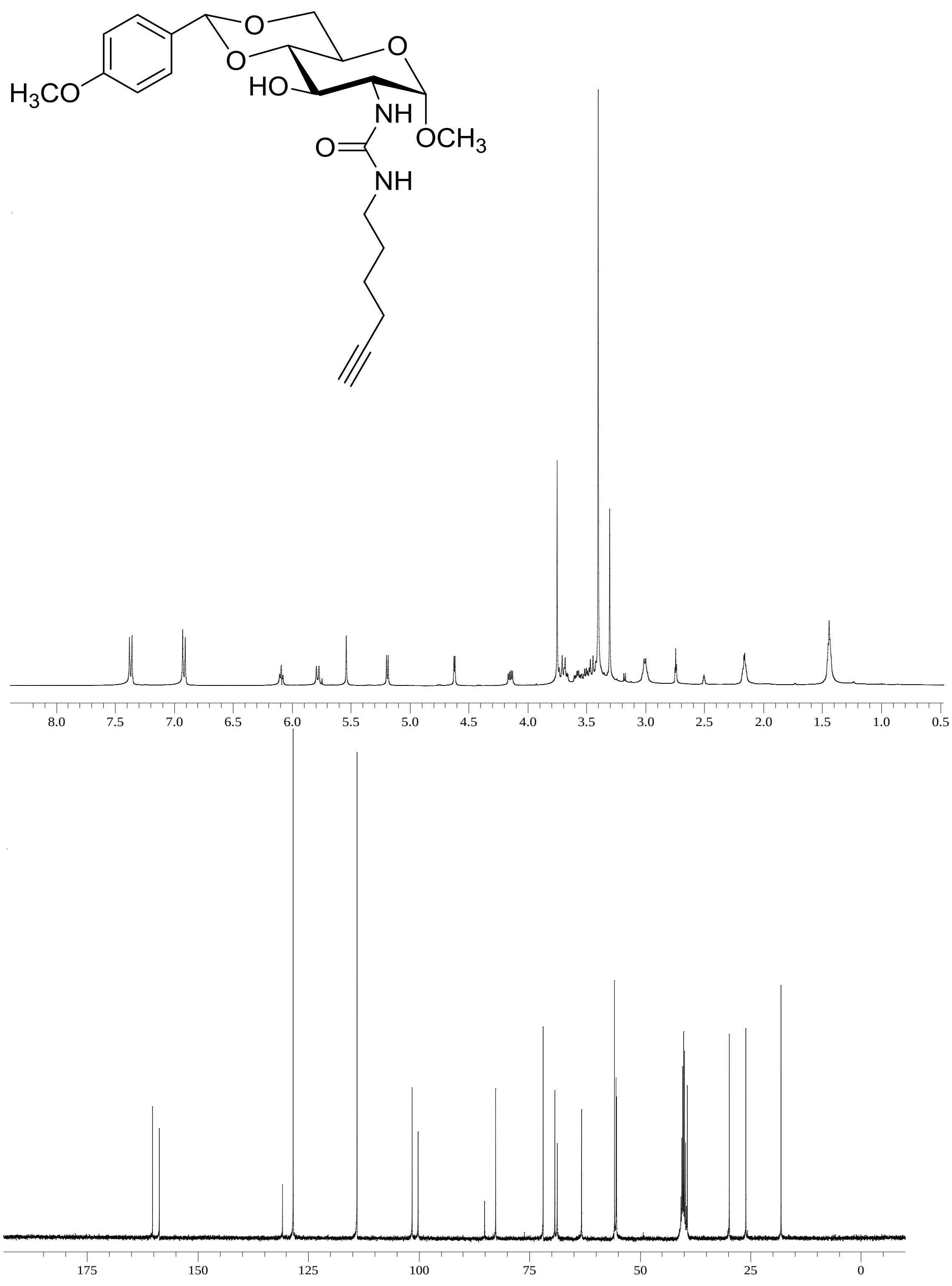

^{1}H and ^{13}C NMR spectra for compound **18**, 400 MHz machine and in DMSO

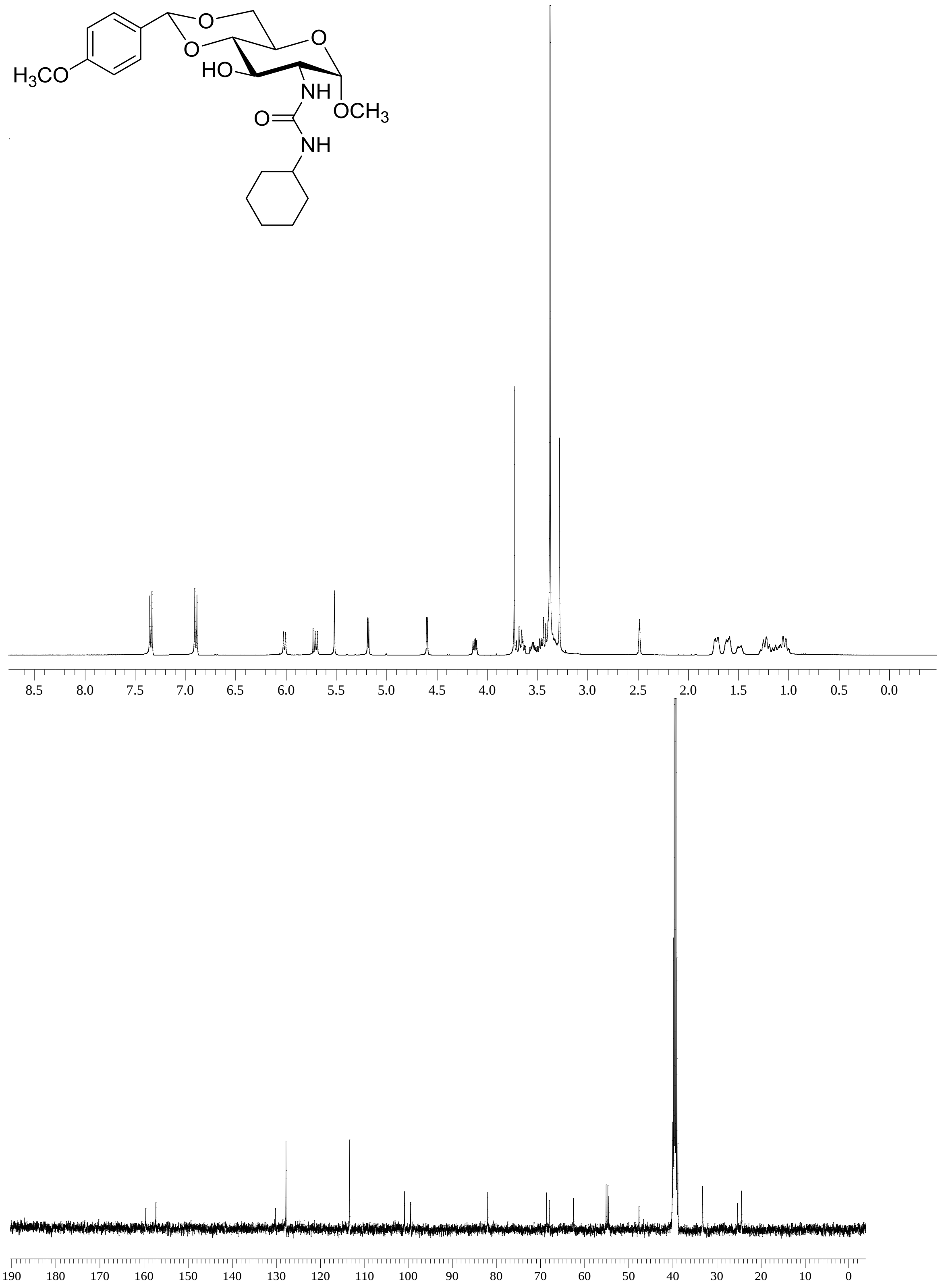

^{1}H and ^{13}C NMR spectra for compound **19**, 400 MHz machine and in DMSO

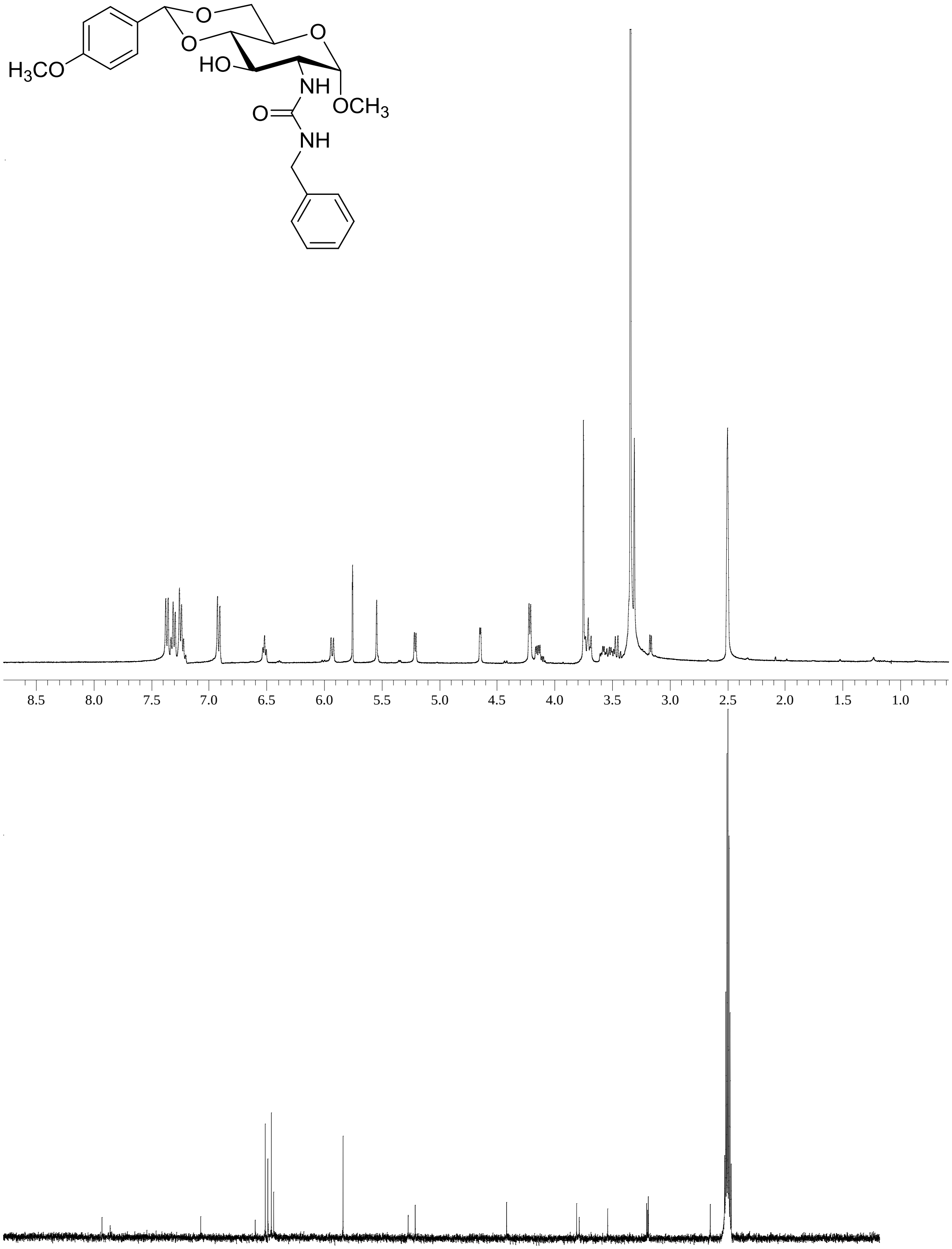

^{1}H and ^{13}C NMR spectra for compound **20**, 400 MHz machine and in DMSO

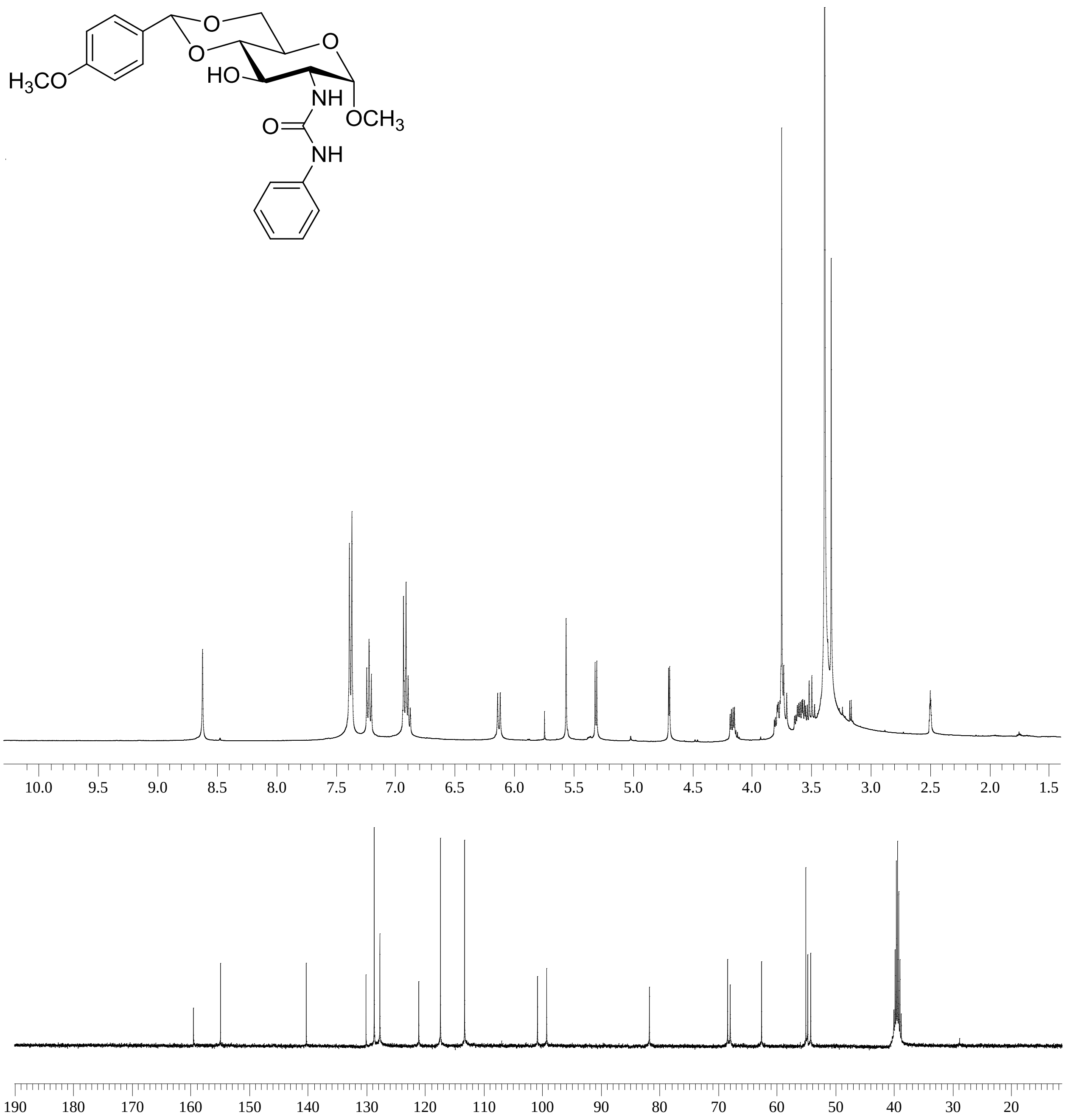

^{1}H and ^{13}C NMR spectra for compound **21**, 400 MHz machine and in DMSO

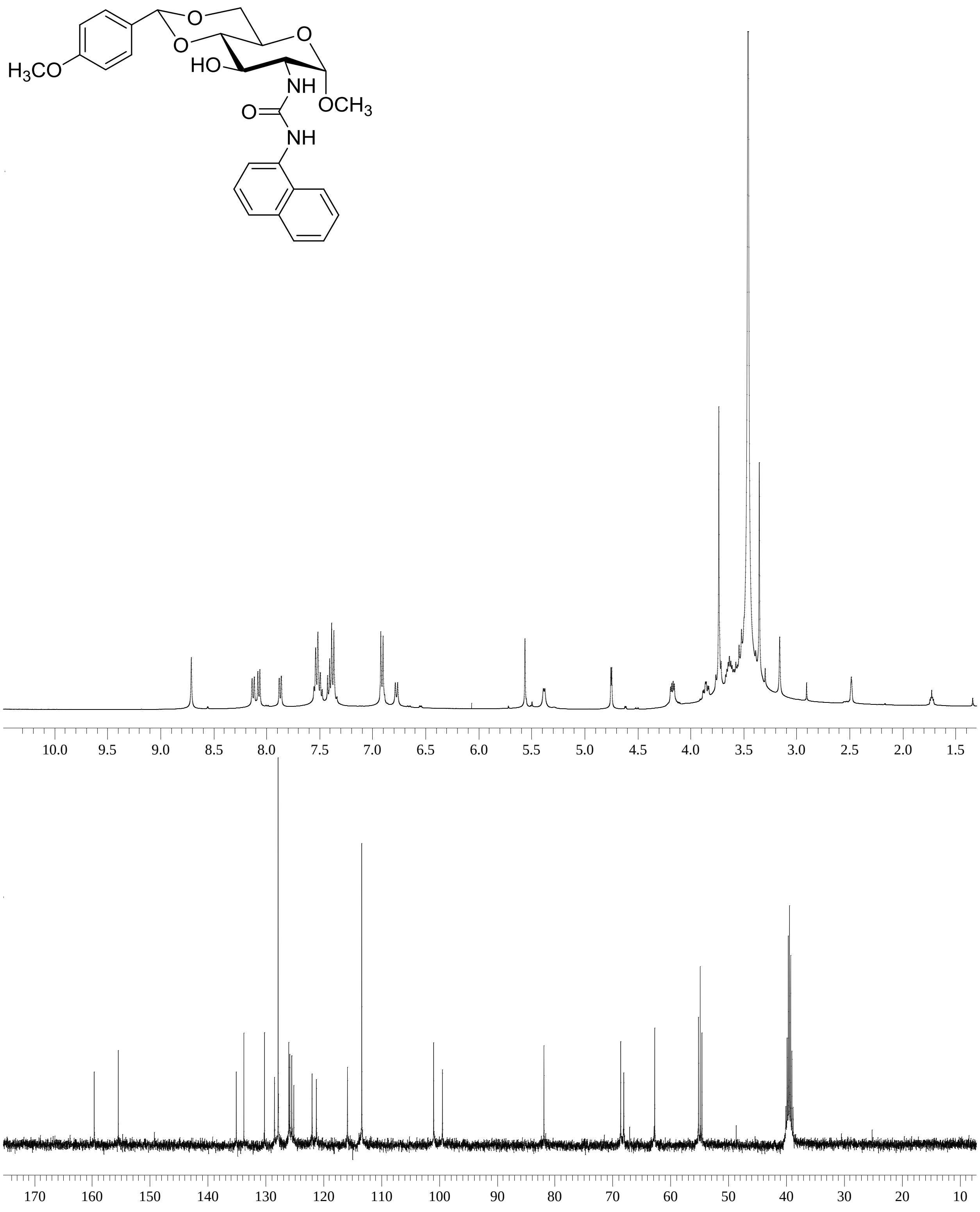

^{1}H and ^{13}C NMR spectra for compound **22**, 400 MHz machine and in DMSO
